# Correlation of Interface Interdiffusion and Skyrmionic Phases

**DOI:** 10.1021/acs.nanolett.3c00428

**Published:** 2023-05-26

**Authors:** Pamela C. Carvalho, Ivan P. Miranda, Jeovani Brandão, Anders Bergman, Júlio C. Cezar, Angela B. Klautau, Helena M. Petrilli

**Affiliations:** † Universidade de São Paulo, Instituto de Física, Rua do Matão, 1371, São Paulo 05508-090, São Paulo, Brazil; ‡ Department of Physics and Astronomy, Uppsala University, Box 516, Uppsala 75120, Sweden; ¶ Laboratório Nacional de Luz Síncrotron, Centro Nacional de Pesquisa em Energia e Materiais, Campinas 13083-970, São Paulo, Brazil; § Faculdade de Física, Universidade Federal do Pará, Belém 66075-110 , Pará, Brazil; ∥ Departamento de Física da Universidade de Aveiro, Aveiro 3810-183, Portugal

**Keywords:** skyrmions, DMI, interdiffusion, symmetric layers

## Abstract

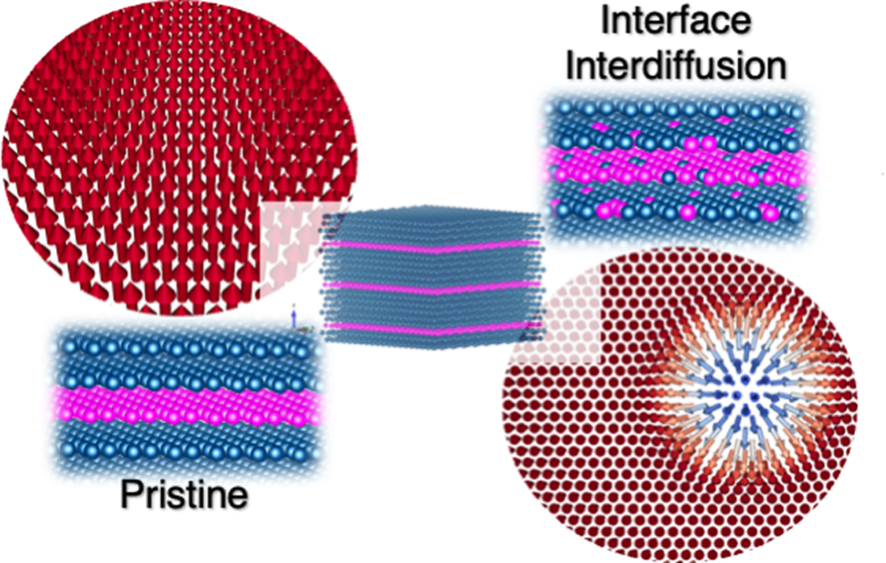

Magnetic skyrmions are prime candidates for the next generation of spintronic devices. Skyrmions and other topological magnetic structures are known to be stabilized by the Dzyaloshinskii-Moriya interaction (DMI) that occurs when the inversion symmetry is broken in thin films. Here, we show by first-principles calculations and atomistic spin dynamics simulations that metastable skyrmionic states can also be found in nominally symmetric multilayered systems. We demonstrate that this is correlated with the large enhancement of the DMI strength due to the presence of local defects. In particular, we find that metastable skyrmions can occur in Pd/Co/Pd multilayers without external magnetic fields and can be stable even near room temperature conditions. Our theoretical findings corroborate with magnetic force microscopy images and X-ray magnetic circular dichroism measurements and highlight the possibility of tuning the intensity of DMI by using interdiffusion at thin film interfaces.

Interface-induced effects on physical properties arising in ferromagnetic/heavy-metal (FM/HM) heterostructures^1^ have been explored as a platform to host a plethora of novel two-dimensional topological magnetic structures such as, skyrmions,^2−5^ skyrmioniums,^6−8^ and bimerons.^9,10^ These chiral magnetic textures are primarily induced by the Dzyaloshinskii-Moriya interaction (DMI),^1,11−15^ which arises in these magnetic systems with intrinsic broken inversion symmetry, combined with strong spin–orbit interaction (SOI) provided by the heavy atoms at FM/HM interfaces.^16−18^

In contrast, zero-field room temperature skyrmions were recently observed in unpatterned symmetric FM/HM/FM multilayers, in particular Pd/Co/Pd,^19−21^ which place these types of structures as prospective candidates for future spintronic devices. Correspondingly, a finite DMI has been experimentally observed in these stackings grown by magnetron sputtering or molecular beam epitaxy (MBE) techniques.^22,23^ Since systems that exhibit inversion symmetry would exclude the DMI, a possible origin of the DMI in sputtered multilayers is an interfacial disorder due to atomic intermixing at the interface,^24−27^ as shown for some symmetrical multilayer stackings.^28,29^ In addition to the DMI, the perpendicular magnetic anisotropy (PMA) in Pd/Co/Pd stacking is also essential to guide the exploration of stable skyrmions.^30−42^ Moreover, lower PMA values was systematically obtained for sputtered samples compared to those grown by MBE (or electron beam evaporation).^43^ It can be noted that a better agreement between the theoretical and experimental PMA data is obtained for MBE-grown samples,^44^ emphasizing that the related *ab initio* calculations considered flat interfaces. Most notably, in unpatterned symmetric multilayers, room-temperature skyrmions were only observed in sputtered samples so far,^19,22^ indicating the relevance of studying the correlation between DMI (and anisotropy) robustness with the interface quality and its influence in favoring the formation of skyrmions.

Here, we combine first-principles calculations based on density functional theory (DFT), using the real-space linear-muffin-tin-orbital within the atomic sphere approximation (RS-LMTO-ASA) method,^45−51^ and atomistic spin simulations (Uppsala Atomistic Spin Dynamics - UppASD)^52−54^ to investigate the microscopic origin of non-negligible DMI and its role in the formation of skyrmions in symmetric multilayers, where we use Pd/Co/Pd stacking as an example. The substantial enhancement of the DMI is explained by the inclusion of atomic defects, where this scenario of local broken symmetry is mainly different from simply considering alloying at the interfaces. The approach used here suggests that the local DMI at the interfaces can be 1 order of magnitude larger than that of the pristine multilayer, depending on the local arrangement of defects. This result is fundamental to understand the experimental findings, obtained by magnetic force microscopy (MFM) and X-ray magnetic circular dichroism (XMCD) measurements, supported by our *ab initio* and spin dynamics simulations, where we show the correlations between interface interdiffusion and the formation of room-temperature skyrmions. Our observations shed light on the perspectives for tuning the DMI strength through the degrees of intermixing in thin film interfaces.

Since multilayer systems are not infinitely thick, a symmetry breaking naturally occurs at their terminating interfaces; in our model, these terminating interfaces are represented by Pd layers. In order to disentangle the surface-induced symmetry breaking from interdiffusion effects, we simulate the Pd/Co/Pd multilayer, without defects, considering two approaches: calculations with semi-infinite geometry (SIG) and infinite multilayers (IM) (inset of Figure 1), where one and five monolayers have been considered for Co and Pd, respectively. The interface exchange coupling (*J*_*ij*_) values and the magnitudes of the Dzyaloshinskii-Moriya vectors  are presented in Figure 1. From Figure 1a, it can be seen that the *J*_*ij*_ values for IM and SIG approaches present very similar behavior. The DMI vectors at the Pd/Co/Pd interface are represented in inset b1 in Figure 1b, where one can see that the vectors present different senses of rotations above and below the Co layer. This indicates a zero net DMI contribution from these atoms, which is directly related to the -DMI anisotropic feature .

**Figure 1:**
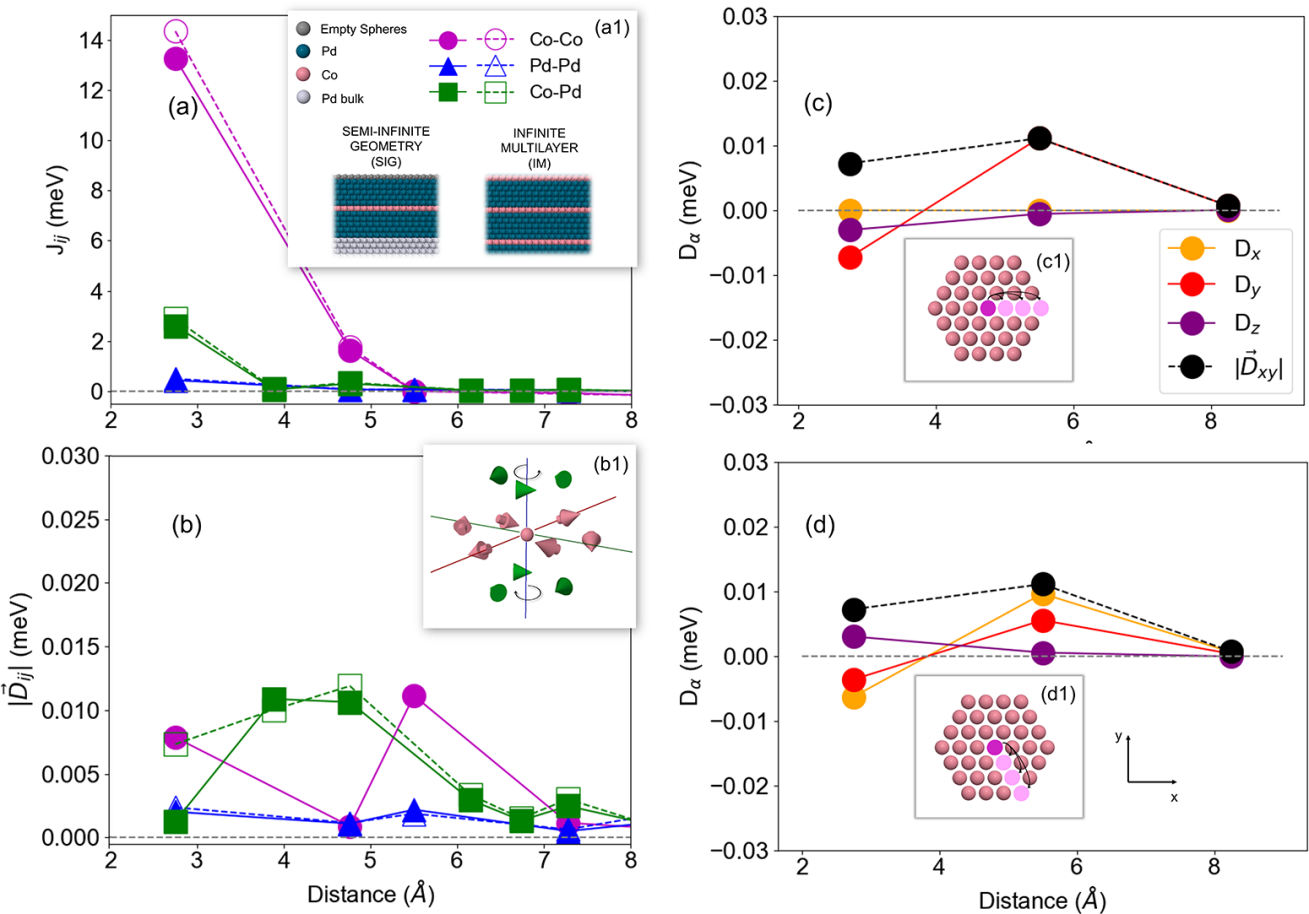
Co–Co, Co–Pd and Pd–Pd interactions at the interface Pd/Co/Pd (a) Exchange coupling (*J*_*ij*_) and (b) DMI strength  as a function of the interatomic distance in the infinite multilayer (solid lines and full markers) and semi-infinite geometry (dashed lines and empty markers). DMI vector components (*D*_α=*x*,*y*,*z*_) and strength of the projected in-plane contribution  for the Co–Co coupling in the semi-infinite geometry, considering the directions (c) [100] and (d) [1–10]. (a1) The real space representation of the semi-infinite geometry and infinite multilayer. (b1) The DMI vectors for Co–Co (pink arrows) and Co–Pd (green arrows) interactions in the semi-infinite geometry for nearest neighbors. The real space representations of the of the Co–Co couplings in the directions (c1) [100] and (d1) [1–10], respectively, in dark and light pink.

Remarkably, when SIG and IM results are compared, the main difference is the IM null Co–Co DMI (not shown in Figure 1b), which arises from the fact that the IM presents inversion symmetry. Although the Pd/Co/Pd interface is also symmetric in SIG calculations, the existence of a vacuum layer breaks the inversion symmetry leading to a small nonzero Co–Co DMI. Thus, a source of the DMI in symmetric multilayers, supposedly with nonbroken inversion symmetry, can be attributed to surface effects, what is expected experimentally. Besides that, we analyze the Co–Co DMI vector components in Figure 1c and Figure 1d, from the SIG system, since it has been suggested in the literature that large in-plane components might be fundamental to the stabilization of skyrmions.^6,55−59^ In fact, the in-plane components () are always larger than the out-of-plane components (); however, the DMI strength is negligible compared to the isotropic exchange coupling. Therefore, since the formation of skyrmions is related to the competition between these interactions, it is unlikely that this DMI is large enough to stabilize spontaneous skyrmions in the ground state or even in metastable states.

We turn now to investigate the effects of interdiffusion on the DMI by performing defect calculations (replacing Co for Pd atoms and vice versa) in the SIG system, considering two cases: a single defect or several defects, in the Co monolayer at the Pd/Co/Pd interface. Here, we define interdiffusion as an asymmetric intermixing of atoms at the interface Pd/Co/Pd, where the amount of Pd and Co defects is not necessarily balanced. The inversion symmetry here is locally broken and the real space illustrations are presented in Figure 2a and Figure 2e, respectively, whereas the plot of the DMI strengths are shown in Figure 2b and Figure 2f. The DMI shows enhanced values for a few Co–Co pair-interactions near the source of the symmetry breaking, while for more distant pairs it tends to the pristine SIG value, indicating a local characteristic of interdiffusion effects on the DMI. Also, as the atoms *i* and *j* are closer to one or more atomic defects, the DMI strength becomes larger, as can be seen in Figure 2f. This is important to illustrate that the defects impact in the DMI behavior is only local and a considerable percentage of interdiffusion can be necessary in order to increase its strength macroscopically. Figure 2c and 2d present the plot of  and , respectively, for the single defect, and analogously, the results for the several defects case are shown in Figure 2g and h. By comparing these plots with the DMI strength, note that while the strongest interactions DMI vectors are out-of-plane for the single defect, in the several defects case, there are many couplings which have a large in-plane DMI vector component and a similar behavior has been found for Co–Co intralayer interactions at Pd layers (Figure S10 in Supporting Information II.B).

**Figure 2:**
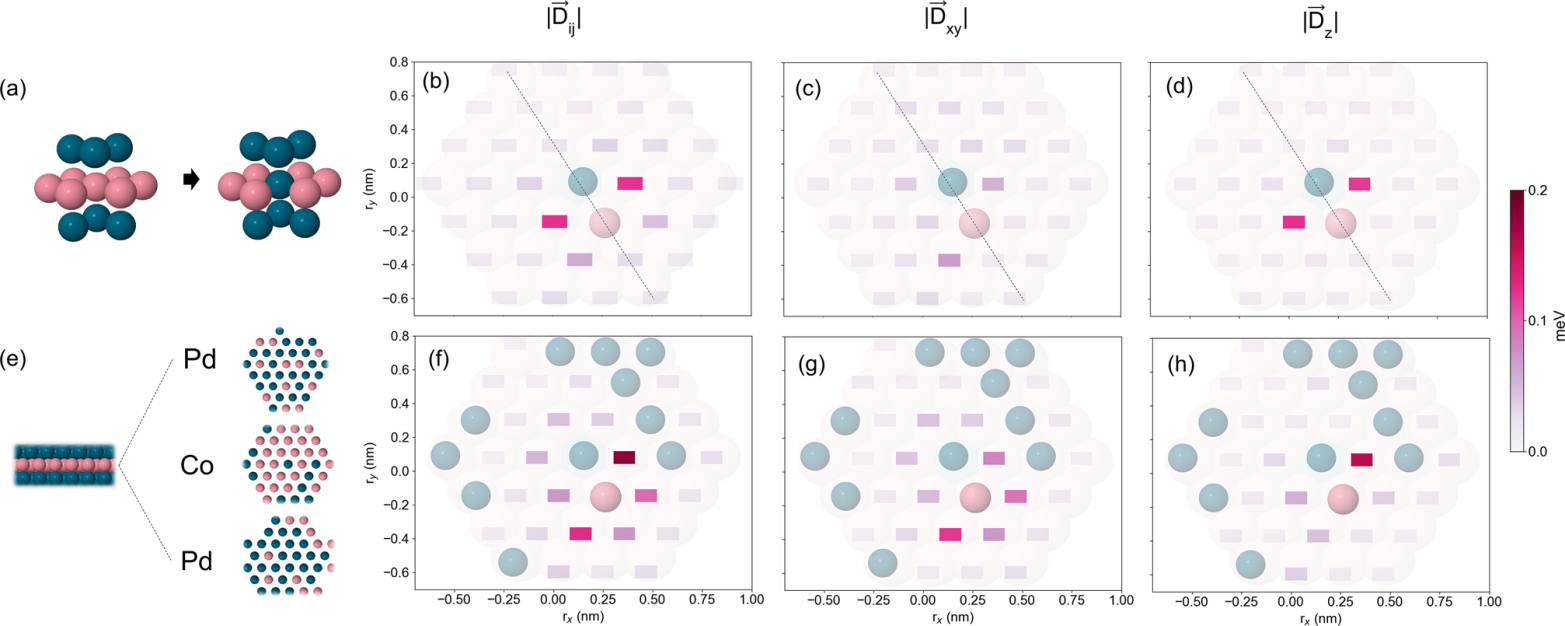
Real space representation with Co atoms in pink and Pd in blue for (a) a single defect at the Co monolayer and (e) several defects, where Co (Pd) atoms are replaced by Pd (Co) atoms at the interface Pd/Co/Pd. Co–Co DMI strength () in the Co monolayer (r_*x*_ and r_*y*_) for the cases with (b) a single defect and (f) several defects. Norm of the projected in-plane DMI vector () for (c) a single defect and (g) several defects. Absolute value of the DMI vector *z*-component () for (d) a single defect and (h) several defects. The pink colormap represents the DMI coupling (squares) between the fixed Co atom (pink in b and f) and the Co atom in each site. Here, the dashed black line is a guide to the eyes and represents a symmetry axis for the interactions.

In order to better compare the pristine and single defect cases, we plot in Figure 3a the norm of the DMI vectors in the system with a single defect, in addition to the pristine SIG results. By comparing the largest DMI from the defect with the pristine case, it is noteworthy to see that the presence of a single defect can increase the coupling by a factor of ∼16. In Figure 3b, we plot the average nearest-neighbor (NN) DMI strength of Co–Co pairs as a function of the number of NN defects (represented by the inset in Figure 3b). We observe an increase in the DMI strength with the number of NN defects since the closer the Co–Co pair is to the defect, the largest is the DMI. This is consistent with what has been observed for the several defects case (Figure 2f). Therefore, we have shown that the DMI is largely affected by defects due to local symmetry breaking with a huge strength enhancement. Since the sign of the DMI differs from the Pd/Co and Co/Pd interfaces it can not be expected that the net effect of interdiffusion is simply additive, i.e., the resulting DMI enhancement should on average be lower than what the single defect indicates. In fact, given a symmetric interdiffusion scenario, it can be argued that the net DMI should be zero, where a random distribution of defects may lead to DMI vectors with random directions (Figure S7 in Supporting Information II.B). However, contrary to this possibility, our extended defect calculation which goes beyond the single-defect picture, presents a significant enhancement of the net DMI in addition to a large number of couplings with expressive in-plane components, indicating favorable conditions to the existence of skyrmions.

**Figure 3:**
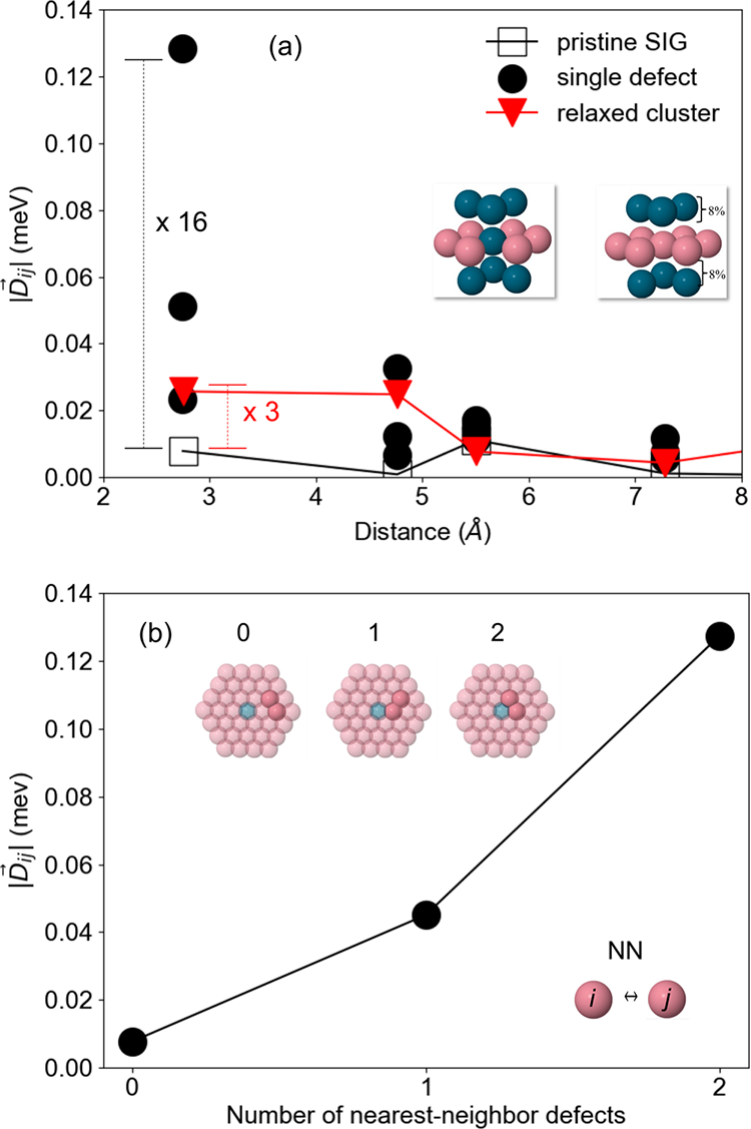
(a) Co–Co DMI strength for the cases: semi-infinite geometry with a single defect (full black circles), considering the site *i* or *j* in the first shell of the defect neighbors; relaxed cluster without defects (red full triangles); and pristine semi-infinite geometry without structural relaxation effects (black open square); see insets. For the single defect, there are three nonequivalent Co–Co pairs, with a symmetry axis defined by the dashed line in Figure 2b (for more details, see Supporting Information II.B). The ratio between the nearest-neighbor DMI in the defect (relaxed cluster) and the pristine system is indicated in black (red); (b) Co–Co DMI strength (nearest-neighbor average) as a function of the number of nearest-neighbor defects (see inset).

Structural relaxation effects have also been investigated, where the Pd atomic positions (NN of a reference Co atom), in Pd/Co/Pd SIG system, were relaxed inward by ∼8% in the out-of-plane direction (Supporting Information I.A). Although the DMI is enhanced due to structural relaxations, the effect is smaller compared to interdiffusion cases (Figure 3a) and the DMI remains almost unchanged (data not shown) by relaxing the Pd/Co/Pd interface with a single defect, indicating that structural relaxations do not seem to play a major role in the DMI strength in the presence of atomic disorder. We also note here that a disordered arrangement of defects is important to the formation of noncollinear nanostructures, which is mainly different than considering an ordered alloy at the interface (Supporting Information I.A).

In addition to the competition between isotropic exchange and DMI, the magnetic anisotropy is also important for the stability of skyrmion structures. We calculated (Supporting Information I.A) the MAE constant (*K*) and an out-of-plane easy axis (PMA) was obtained, for both pristine SIG and IM cases (Table S1 in Supporting Information II.C), where the value obtained for the IM is 0.23 meV/atom. The magnetocrystalline anisotropy obtained for the IM is consistent with other theoretical works in the literature,^32,42^ while it is much larger than the experimental anisotropy obtained from sputtered samples with similar Co thickness.^36,41^ Nevertheless, as we mentioned, the experimental PMA for this system is known to be very sensitive to different deposition methods.^30−41^

From the J_*ij*_ and  results (Table S1 in Supporting Information II.B), we note that although the presence of defects at the interface Pd/Co/Pd largely affects the DMI behavior, the *J*_*ij*_ couplings are not significantly affected. Since, contrary to *J*_*ij*_, both the DMI and the magnetic anisotropy can depend strongly on how the system is synthesized, we now turn toward evaluating the dependence of chiral-type structure emergence, as a function of DMI strengths and anisotropy values. We do this by calculating the zero external magnetic field and zero temperature phase diagram for the Pd/Co/Pd multilayer via the Monte Carlo approach (or the 4-IIS method; see Supporting Information I.B), where the obtained magnetic configuration is further relaxed with atomistic spin dynamics simulations. The computed phase diagram is shown in Figure 4, where we used the *J*_*ij*_ couplings from the pristine SIG and we varied (*i*) the out-of-plane effective anisotropy strength (*K*_eff_), which includes the shape anisotropy (Supporting Information I.A), and (*ii*) the DMI strength with a scaling DMI factor, ϵ^DMI^ (, Supporting Information I.B).

**Figure 4:**
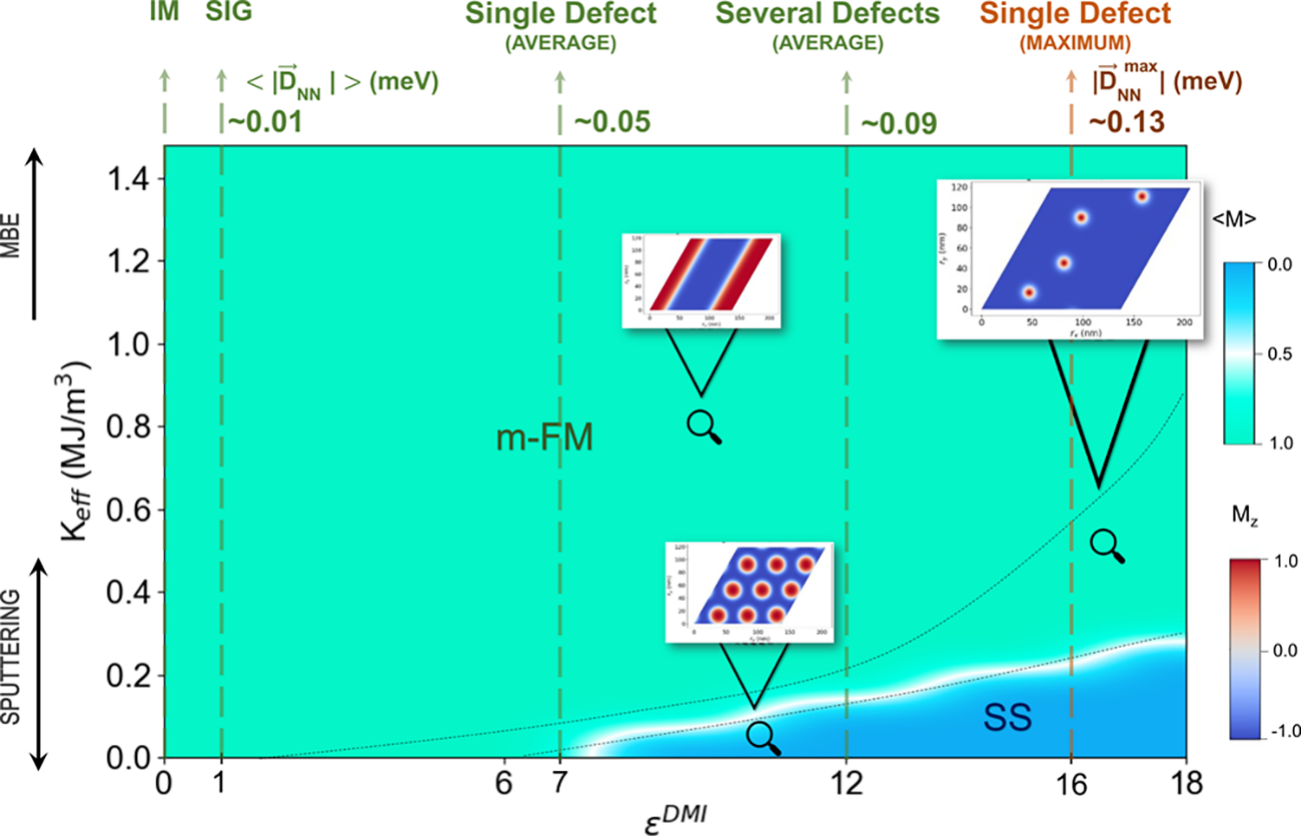
Phase diagram of the Pd/Co/Pd multilayer, at *T* = 10^–4^ K, where the effective anisotropy strength (*K*_eff_) and scalar DMI factor (ϵ^DMI^) are varied. The spin spiral (SS) and monodomain-FM (m-FM) ground state phases are shown, and the snapshots indicate regions where we obtained metastable states: (*i*) stripes, for large magnetic anisotropy; (*ii*) isolated skyrmions (dashed black lines); (*iii*) skyrmions lattices or skyrmions mixed with stripes, for large DMI strengths and small anisotropies. The m-FM corresponds to normalized average magnetization ⟨M⟩ = 1 (green), the SS corresponds to ⟨M⟩ = 0 (blue), and the transition region (white) ⟨M⟩ = 0.5. The colormap of the snapshots refers to the *z*-component (M_*z*_) of the spins. The green and orange dashed lines indicate the average and maximum nearest-neighbor (NN) Co–Co DMI strength (in meV, where the values are rounded to the second decimal digit), respectively, with its corresponding ϵ^DMI^ for the infinite multilayer (IM), semi-infinite geometry (SIG), a single, and several defects. The experimental anisotropy ranges using different deposition methods, found in the literature, are also indicated in black arrows (for MBE the values can be even larger). Finally, the *K*_eff_ values in the *y*-axis were converted from meV/atom to MJ/m^3^ according to the atomistic definition (Supporting Information II.C).

First, there is a correlation between the number of repetitions of metallic stacking and an increase in the DMI strength,^60^ which further support the use of the scalar DMI factor in the phase diagram. Besides that, the factor ϵ^DMI^ should not be seen as a simple extrapolation of a single defect result but as a reasonable initial assumption, since the calculation of many Pd defects in the Co layer resulted in an average DMI magnitude enhancement of ϵ^DMI^ ∼ 12 for NN interactions (Figure 4). For ϵ^DMI^ = 1, which corresponds to the *ab initio* Co–Co pristine SIG DMI value, the ground state is monodomain-FM (m-FM) for all values of anisotropy considered here. Since skyrmions are observed experimentally for Pd/Co/Pd, these theoretical simulation conditions do not reproduce the experimental results, and we can foresee that the DMI in the sputtered Pd/Co/Pd samples is larger than the pristine DFT predicted. We suggest that the lack of intermixing of atoms at the interface (which largely affects the DMI, as already shown here) produces this discrepancy between theoretical (pristine case) and experimental results.

From the phase diagram in Figure 4, we see that for most combinations of magnetic anisotropy and DMI strength, the ground state is m-FM (green region), while a noncollinear ground state (blue region) is feasible only for small values of magnetic anisotropy and large DMI strengths. Metastable states are indicated by snapshots and although these skyrmionic configurations can only be here found for ϵ^DMI^ ≥ 1.5 (dashed line), note that considering the experimental PMA (*K*_eff_ = 0.06 MJ/m^3^) measured in the sample where the skyrmions were observed,^19^ ϵ^DMI^ must be even larger. Here, the use of ϵ^DMI^ is an approximation where the DMI vector directions are fixed from the pristine results. To investigate how this could affect the formation of the observed isolated skyrmions, we have also performed spin dynamics simulations for two random DMI models, inspired by the Edwards-Anderson model for spin glasses:^61,62^ (i) fully random and (ii) partially random. While in model (i), the DMI vectors present a spherically symmetric distribution, for model (ii), the presence of defects is treated as perturbations in the pristine Co–Co DMI couplings, where its random distribution is constrained by DFT outcomes. Our results indicate that the stabilization of metastable skyrmionic states is still possible for model (ii).

The anisotropy also plays an important role in the determination of the ground state. For low anisotropy values, the DMI should be at least a factor of 6 larger than the pristine case in order to originate a spin spiral (SS) ground state, while for the range of anisotropy from MBE-grown samples the ground state is always m-FM. Then, in order to Pd/Co/Pd multilayers host skyrmions, the anisotropy should be small (samples grown by magnetron sputtering), and the DMI should be larger than the pristine *ab initio* values. This agrees with experimental results, where the skyrmions in these multilayers have been observed only in sputtered samples. Here, we indicate the interdiffusion as a mechanism which acts on two different properties simultaneously favoring the formation of noncollinear nanostructures in Pd/Co/Pd multilayers by lowering the anisotropy^43^ and increasing the DMI strength.

We complement our hitherto theoretical study with experiments on the same system. Here, the Pd/Co/Pd multilayers were grown, similar to previous work,^19^ by magnetron sputtering with Pd thickness of 1 nm and Co thickness of 0.2 nm (resulting in thickness similar to the one used in the first-principles calculations). Note that experimentally the trilayer Pd/Co/Pd was repeated 15 times, while we have only considered a single repetition in the theoretical calculation. The magnetic nanostructures were observed with magnetic force microscopy images, and to explore the multilayer magnetic properties, we performed here X-ray absorption spectroscopy (XAS) and X-ray magnetic circular dichroism (XMCD). Figure 5a shows the X-ray absorption spectra obtained around the L_2,3_ edges for the Co 0.2 nm thick sample, and Figure 5b shows the respective XMCD (difference between right and left circularly polarized X-ray absorption). In order to obtain the theoretical magnetic moment at room temperature, we performed a combination of first-principles and atomistic spin dynamics calculations (Supporting Information II.D). The sum of spin and orbital magnetic moments per Co atom (0.92 ± 0.03 μ_B_), inferred from the results presented in Figure 5b, and the theoretical spin magnetic moments at 300 K (0.83 ± 0.11 μ_*B*_/atom) presents an excellent agreement, indicating that we can expect that our calculations provide realistic values for the magnetic *J*_*ij*_ couplings.

**Figure 5:**
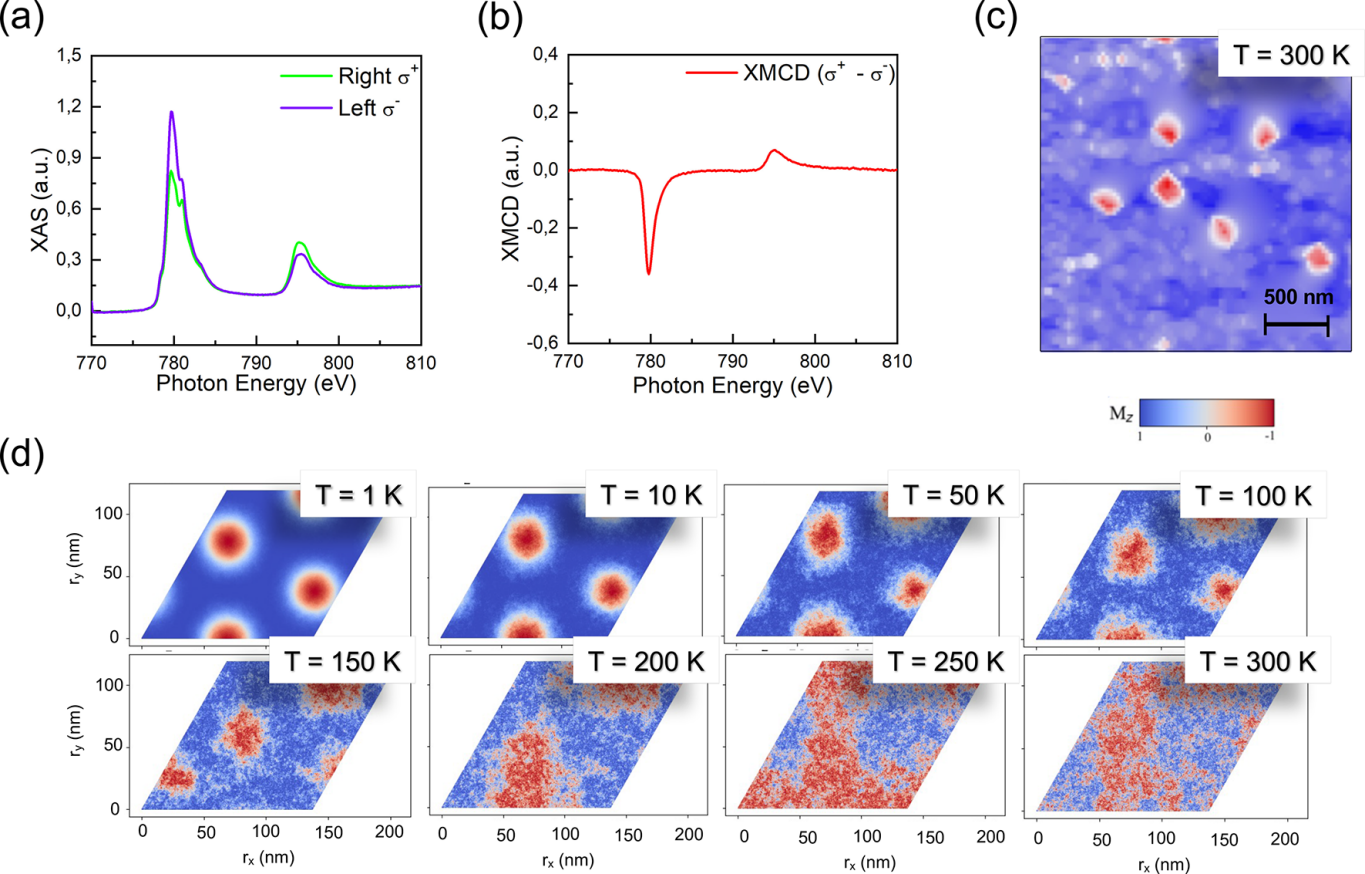
(a) X-ray absorption spectroscopy and (b) X-ray magnetic circular dichroism obtained at room-temperature around L_2,3_ Co edges; (c) magnetic force microscopy images for the Pd/Co(0.2 nm)/Pd at room temperature (showing magnetic features in nanoscale), and (d) snapshots of the calculated (using ϵ^DMI^ = 10 and *K*_eff_ = 0.15 MJ/m^3^, converted using the atomistic definition) metastable skyrmion lattice for different temperatures.

Concerning the spin texture for this system, in Figure 5c, we show the observed magnetic images for the Pd/Co (0.2 nm)/Pd sample. MFM image shows the formation of isolated skyrmions in a monodomain background at room-temperature and zero-field. This result suggests the formation of skyrmions as “local excitations” on the sample, which agrees with our simulations in Figure 4. In order to compare, at room temperature, theoretical calculations with experimental images, considering a PMA value close to the experimental one (*K*_eff_ ∼ 0.1 MJ/m^3^), here, we have chosen a skyrmionic configuration corresponding to ϵ^DMI^ = 10. We turn now to the temperature-dependent evolution of the skyrmions stability for this magnetic configuration in the time interval analyzed by spin dynamics simulations (50 ps), as shown in Figure 5d. At *T* = 0 K, a skyrmion lattice is a metastable state, and the Berg-Lüscher invariant^63^ is preserved up to *T* = 150–200 K. On the other hand, further raising of the temperature annihilates the skyrmions, changing the Berg-Lüscher invariant in a sizable amount of time. The fact that theoretical skyrmions in Figure 5d are stable at temperatures lower than the one observed experimentally (Figure 5c), can be in part justified by the number of Pd/Co/Pd trilayer repetitions used in the experimental sample, which is known to enhance thermal stability.^18,64^

In this work, we investigate the formation of skyrmions and their correlation with interdiffusion effects and perpendicular magnetic anisotropy in Pd/Co/Pd multilayers through a combination of theoretical and experimental methods. In the simulation of interfacial interdiffusion, a significant enhancement of the DMI strength has been obtained due to the presence of random defects, which favors the emergence of metastable skyrmionic states, considering anisotropy values from sputtered samples. Also, this strong DMI strength, induced by atomic disorder, leads to a high-temperature skyrmion stability, on the order of magnitude of experimental results. It should be noted that such disorder is not equivalent to considering uniform alloying at the interfaces. The approach put forward here might also apply to other unpatterned symmetric FM/HM/FM multilayers. For that, as proposed here, a local scenario description would be relevant for the simulations involving an effective spin Hamiltonian and crucial to the interpretation of room temperature skyrmion measurements. Therefore, our work contributes to clarify one possible microscopic origin of the skyrmion formation in unpatterned symmetric stackings. Also, the present first-principles calculations overcome known difficulties in the currently available calculations of DMI when considering the effects of realistic conditions such as atomic disorder. This study opens up new perspectives to tune the DMI in disordered systems in order to create topological magnetic materials, which are essential ingredients for spintronics applications.

## Supporting Information Available

Detailed description of the theoretical methodology used here (first-principles and atomistic spin dynamics) and presentation of other theoretical results that further support the main findings of this work; the experimental procedure to obtain the magnetic moments from X-ray absorption spectroscopy (XAS) and X-ray magnetic circular dichroism (XMCD) is also described (PDF)
