# Supplementary material for: Correlation of Interface Interdiffusion and Skyrmionic Phases
Source: Nano Lett. 2023 May 26;23(11):4854–61. doi: 10.1021/acs.nanolett.3c00428 (PMC10273311; doi:10.1021/acs.nanolett.3c00428)
Supplement: Supplementary file 1 — nl3c00428_si_001.pdf [file nl3c00428_si_001.pdf]

Supporting Information to *Correlation of interface interdiffusion and skyrmionic phases*

Pamela C. Carvalho,[†] Ivan P. Miranda,[‡] Jeovani Brandão,[¶] Anders Bergman,[*,‡] Júlio C. Cezar,[¶] Angela B. Klautau,[*,§,‖] and Helena M. Petrilli[†]

†*Universidade de São Paulo, Instituto de Física, Rua do Matão, 1371, 05508-090 São Paulo, SP, Brazil*

‡*Department of Physics and Astronomy, Uppsala University, 75120 Box 516 Sweden*

¶*Laboratório Nacional de Luz Síncrotron, Centro Nacional de Pesquisa em Energia e Materiais, 13083-970, Campinas, SP, Brazil*

§*Faculdade de Física, Universidade Federal do Pará, CEP 66075-110, Belém, PA, Brazil*

‖*Departamento de Física da Universidade de Aveiro, 3810-183 Aveiro, Portugal*

E-mail: anders.bergman@physics.uu.se; aklautau@ufpa.br

I. Computational details

A. *Ab initio* simulations

We used the real-space linear-muffin-tin-orbital within the atomic sphere approximation (RS-LMTO-ASA) method,[1–7] based on the Density Functional Theory (DFT),[8] to perform fully relativistic first-principles calculations and obtain the electronic structure of Pd/Co/Pd multilayers. The Haydock recursion,[9] with the recursion cut-off of $LL = 31$, in addition to the Beer-Perttifor terminator[10] was employed to obtain the ground state electronic density. The local spin density approximation (LSDA) by von Barth and Hedin[11] was used as the

exchange-correlation functional. Since the code is implemented in the real-space, this method presents advantages when treating systems with broken inversion symmetry, such as semi-infinite geometries, impurities and defects calculations. The exchange coupling (J_{ij}) and Dzyaloshinskii-Moriya vector ($\vec{D}_{ij}$), between atomic sites i and j located at the semi-infinite geometry (SIG), or infinite multilayer (IM), were calculated for a ferromagnetic configuration using the RS-LMTO-ASA code.[1,12,13] Since the J_{ij} and $|\vec{D}_{ij}|$ values can be different for (i, j) pairs of atoms in the same neighboring shell, we considered the average for each interatomic distance. Regarding the $\vec{D}_{ij}$ calculation we note, however, that several ways have been proposed to evaluate its magnitude theoretically, and a broad discussion can be seen in Ref.[14] and references therein; and the method used here is derived from the multiple scattering electronic structure theory as implemented in the RS-LMTO-ASA code in Ref.[13]

Here, we used the force theorem (FT) to obtain the magnetocrystalline anisotropy energy (MAE), by performing single iterations of non-collinear calculations with the magnetic moments aligned in different directions on top of a previously converged scalar-relativistic charge densities. The MAE is defined as the band energy difference between out-of-plane and in-plane magnetization and we assume that only the Co monolayer is contributing to the MAE. Here, we rotated the magnetization axis in the xz plane (z is the perpendicular-to-plane direction), from in-plane ($\theta = 0°$) to out-of-plane ($\theta = 90°$). The energy is re-scaled, so the minimum value is set to zero. We also fitted the curve using the following equation, typical for the uniaxial magnetocrystalline anisotropy disregarding higher-order terms,

$$E = K \cos^2(\theta + \theta_K), \tag{1}$$

where θ (θ_K) is the angle between the magnetization axis (easy-axis) and the xy plane, and K is the MAE constant. To obtain the effective magnetic anisotropy (K_{eff}), we subtract the dipole-dipole contribution (E_{dip}) from the MAE.

The MAE results obtained here are corroborated by $\vec{k}$-space calculations with the Quan-

tum ESPRESSO (QE) package,[15,16] also making use of the FT.[17] Past experiences have established the standard Perdew, Burke, and Ernzerhof (PBE) parametrization[18] of the generalized gradient approximation (GGA) as a good choice of exchange-correlation functional for structural properties of transition-metal systems. Therefore, in QE, PBE-GGA was employed in its modified version, known as PBEsol.[19] Analogously to the RS-LMTO-ASA procedure, the FT calculations were performed in two steps: (*i*) first, a self-consistent calculation with ultrasoft scalar-relativistic pseudopotentials (without spin-orbit coupling) to obtain the charge density and the spin moment allocation in real space; and (*ii*) a non-self-consistent calculation with ultrasoft full-relativistic pseudopotentials (with spin-orbit coupling) where the spin moments are globally rotated to a given direction. The MAE, then, is defined as the difference of the band energies obtained from two independent step-(*ii*) calculations with distinct magnetization directions. For the steps (*i*) and (*ii*) we used $30 \times 30 \times 10$ and a denser $50 \times 50 \times 10$ $\vec{k}$-point meshes, respectively, in the Monkhorst-Pack scheme.[20] The Marzari-Vanderbilt cold smearing[21] with 0.01 Ry broadening width was used with plane-wave kinetic energy cutoffs of 90 Ry (wave functions) and 900 Ry (charge density). The MAE was calculated following 3 structural models, all initially set as a 2×2 IM with 5 layers of Pd and 1 layer of Co (24 atoms in total), in a perfect fcc(111) arrangement and the experimental lattice parameter of bulk Pd, $a_{\mathrm{Pd}} \sim 3.89\,\text{Å}$, as indicated by experimental results. The 3 structural IM models were constructed as: without any relaxation, partial relaxation (*i.e.*, only the Co layer and the two Pd adjacent layers), and full relaxation (including positions and lattice parameter) in the variable cell optimization algorithm. The models with structural optimizations resulted in inward Co-Pd distance relaxations of 6.7% (partial relaxation) and 8% (variable cell), for which we considered the upper limit in relaxation tests in real-space; in the variable cell calculation, the optimized lattice parameter is $3.83\,\text{Å}$, representing a decrease of $\sim 1.6\,\%$ w.r.t. the experimental value.

In the RS-LMTO-ASA context, the Pd/Co/Pd multilayers were investigated through the simulation of a cluster of atoms ($\sim 20,000 - 33,000$) in the direction [111], with a pristine

fcc structure, lattice a_{Pd} and following an ABC-ABC stacking. This system was constructed following two approaches: (a) SIG and (b) IM calculations. For (a), the stacking is composed by 1 layer of atoms represented by empty spheres to mimic vacuum; 5 layers of Pd atoms below the empty spheres; 1 layer of Co atoms adjacent to the Pd layers and 5 layers of Pd atoms below the Co layer. The remaining atoms of the cluster, below the Pd layers included in the self-consistent calculation, has its properties fixed with the potential parameters from Pd bulk. We note here that while the experimental trilayer has been repeated 15 times, SIG calculations can be computationally costly and it is not feasible to replicate the exact experimental setup in the calculations. Here, the inclusion of one layer of empty spheres is needed in order to treat the charge transfer correctly in semi-infinite geometries, since it provides a basis set to the wave functions in the vacuum region.[22,23] For (b), the stacking follows the same pattern from the SIG, Pd(5 monolayers)/Co(1 monolayer)/Pd(5 monolayers), with the exception that this stacking is repeated and we do not include empty spheres. It is important to note that the thickness of each element was chosen according to the experimental setup (see Experimental Details).

In order to simulate the system with a single defect, we used the converged electronic structure from the SIG approach and re-performed self-consistent calculations for a small cluster of atoms (111), at the Pd/Co/Pd interface, where one Co atom is replaced by Pd. Here, this set includes 37 Co atoms and 37 Pd atoms above and below the Co monolayer. The rest of the cluster, which is not included in the self-consistent calculation, remains with its SIG electronic structure fixed. Analogously, for several defects, we used the same small cluster and randomly selected atoms to be defects in the three layers. We notice that defects in the Pd (Co) layer are Co (Pd) atoms. It should be noted that in the limit of a fully symmetric and homogeneous interdiffusion scenario, the inversion symmetry would be restored globally but not locally.

To investigate the possibility of larger percentages of interdiffusion, ordered alloys were calculated, considering the IM approach. Here, we followed the same procedure adopted in

the IM, with the exception that we considered an ordered intermixing of atoms only at the Pd/Co/Pd interface. A balanced asymmetric interdiffusion is simulated through the 25% ordered alloy (Fig. 1(a)), where only the Co/Pd interface presents intermixing of atoms, with a concentration of 25% of Pd (Co) atoms in the Co (Pd) layer. On the other hand, a balanced symmetric interdiffusion is simulated through the 50% ordered alloy (Fig. 1(b)), where both the Pd/Co and Co/Pd interfaces present intermixing of atoms, with a concentration of 50% of Pd atoms in the Co layer and 25% of Co atoms in the Pd layers. To simulate conditions that could represent scenarios where the interdiffusion is globally symmetric, we have performed spin dynamics simulations for the ordered alloys. Even though, the DMI results are indeed larger compared to the pristine SIG and locally as large as the defect cases, the simulations indicate that only monodomain FM (m-FM) states are stable in these systems at the interface.

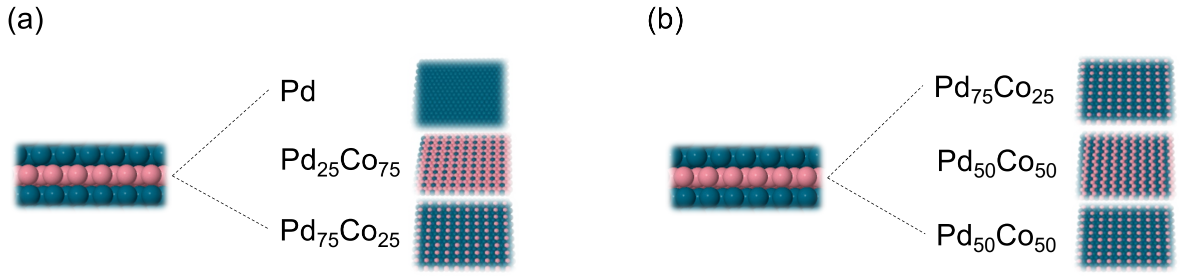

Figure 1: Real space representation of the (a) 25% and (b) 50% ordered alloy configurations calculated as a IM. The blue (pink) spheres represent Pd (Co) atoms.

To investigate the DMI behavior in the presence of structural relaxations, here, we used an approximation where the self-consistent procedure in the RS-LMTO-ASA method was re-performed considering a displacement of $\sim 8\%$ only for nearest-neighbors Pd atoms around a reference Co atom in the SIG cluster (with 43 atoms). As this approximation locally decreases the symmetry of the system, it may induce a larger DMI. Therefore, the effects observed on the DMI come both from the hybridization of Co-Pd states (due to the displacement of atoms) and the locally induced break of inversion symmetry. Other tests have been conducted

on the IM, where the symmetric displacement (by $\sim 8\%$) of the entire Pd layer above and below the Co layer does not affect the DMI, which is still null. However, an asymmetric displacement, where only one of the Pd layers is relaxed inwards, induces a non-null DMI, as expected.

B. Spin dynamics simulations

The spin dynamics simulations were done using the Uppsala Atomistic Spin Dynamics (UppASD)[24–26] code. To obtain the magnetic configuration of the ground-state, we first performed a Simulated Annealing (SA) optimization, starting with a temperature larger than the Curie temperature (T_C) and gradually decreasing it to $T \sim 0$ K, with each step of the cooling schedule thermalized with 2×10^4 Metropolis Monte Carlo (MC) iterations. Particular attention was given to the low temperature range, where we considered a ΔT interval of $4 - 20$ K below $T = 50$ K in the SA process.

Although the Markov chain Monte Carlo methods (such as SA) are established, the problem of reaching the ground-state in spin systems becomes particularly hard to be treated when multiple local minima with accessible energy barriers are present in the solution space. Roughly speaking, such local minima correspond to metastable noncollinear configurations, which can be, among other causes, introduced by magnetic frustration,[27,28] and chiral interactions, such as DMI. One possible way to overcome this difficulty is to make use of more efficient methods to solve hard combinatorial optimization problems, such as the population-annealing Monte Carlo,[29] or the genetic algorithm.[30,31] Another reasonable way is to impose initially good candidates for the ground-state in each external field and temperature (B_{ext}, T) conditions – based on symmetry arguments and/or previous experimental results, for example. Given the layered (2-dimensional) nature and the C_{3v} symmetry of our problem, here we choose initially four imposed states, namely: monodomain-ferromagnetic, paramagnetic, spin-spiral, and skyrmion lattice. The total energies of these four states (after an initial relaxation) were compared with the one obtained with SA, and lowest-energy configuration

was chosen. Henceforth, for sake of conciseness, this method will be referred as the four imposed initial states (4-IIS).

In the spirit of imposing an initial spin-spiral state, the following equation can be used:

$$\hat{e}(\mathbf{r},\mathbf{q}) = \hat{s}\cos(\mathbf{r}\cdot\mathbf{q}) + \hat{n}\sin(\mathbf{r}\cdot\mathbf{q}), \tag{2}$$

where $\mathbf{q}$ is the wave vector, $\hat{e}$ is the local magnetization direction, $\mathbf{r}$ is the real-space spin position and $\hat{s}$ and $\hat{n}$ are unit vectors defining the rotational plane of the spiral. The energy landscape can be mapped out, given the two unit vectors $\hat{s}$ and $\hat{n}$, by calculating all the spin spirals total energies with corresponding $\mathbf{q}$ vectors which are commensurate with the system dimensions. Then, using over-damped zero-temperature atomistic spin dynamics simulations, the $\mathbf{q}$ vector corresponding to the spin spiral with lowest energy is identified and further relaxed. Surely, this lower-energy state will be bounded to our $(\hat{s},\hat{n})$ initial guess, for which we try different unit vector pairs. Same reasoning can be done to construct skyrmion lattices, following the definition of Eq. 2. However, in this case, a $3\mathbf{q}$ vector state is created, *i.e.* a magnetic texture described by $3\mathbf{q}$ vectors with the same magnitude of $\mathbf{q}$ but rotated 120° between them.

After defining the configuration with minimum energy, the Landau-Lifshitz-Gilbert (LLG) equation[25,32,33] is solved to further relax the system:

$$\frac{d\vec{m}_i}{dt} = -\frac{\gamma}{1+\alpha^2}\vec{m}_i \times \left[\vec{B}^i_{\text{eff}} + \frac{\alpha}{m_i}(\vec{m}_i \times \vec{B}^i_{\text{eff}})\right], \tag{3}$$

where $\vec{B}^i_{\text{eff}} = -\frac{\partial\mathcal{H}}{\partial\vec{m}_i} + \vec{b}_i(T)$ is the effective field acting over the i-th site, $\vec{b}_i(T)$ is a stochastic field to consider temperature T effects by using Langevin dynamics,[26] α is the Gilbert damping parameter, and γ is the gyromagnetic ratio. The Hamiltonian used in the determination of the ground and metastable states, and in the LLG equation is given in Eq. 4, where the

J_{ij}, $\vec{D}_{ij}$ and K_i parameters are obtained from RS-LMTO-ASA method:

$$\mathcal{H} = -\sum_{i,j} J_{ij}(\hat{e}_i \cdot \hat{e}_j) - \sum_{i,j} \vec{D}_{ij} \cdot (\hat{e}_i \times \hat{e}_j) + \sum_{i,j} K_i(\hat{e}_i \cdot \hat{e}_j^K)^2 - \sum_i \vec{B}_{ext} \cdot \vec{\mu}_i, \quad (4)$$

where $\hat{e}_i$ and $\hat{e}_j$ are the directions of the respective magnetic moments $\vec{\mu}_i$ and $\vec{\mu}_j$ of sites i and j, and $\hat{e}_i^K$ $(= z)$ is the direction of the magnetocrystalline anisotropy easy-axis. In turn, for some situations, such as the measurement of Co magnetic moment , the Zeeman term was considered in $\mathcal{H}$, being $\vec{B}_{ext}$ the external applied field. In the spirit of the previous discussion, we note here that while the magnetic configuration given by the Monte Carlo approach is not always the proper ground-state of the system, since it is possible to arrive in multiple local minima (metastable states) in the energy landscape, the 4-IIS method generally provides the closest (lower energy) to the ground state of the system. However, if one is searching for metastable states through Simulated Annealing (SA) calculations, it is essential to precisely control the temperature reduction rate such that it induces significant "jumps" in the energy landscape. These "jumps" should be large enough to prevent the system from overcoming the energy barriers that exist in the vicinity of local minima.

In this paper, we considered the interactions J_{ij} and $\vec{D}_{ij}$ between the first five shells of $3d-3d$ neighbors. We performed the simulations with a 500×500 spin lattice and – as we are not interested in the determination of realistic time intervals of dynamical processes (such as the skyrmion lifetimes) – a large damping parameter ($\alpha = 0.5$), in order to accelerate the relaxation. Also, some tests were performed for the 800×800 lattice, giving the same output. Here, only the Co layer is simulated, since the influence of Pd atoms is indirectly accounted in the calculation of the J_{ij} and $|\vec{D}_{ij}|$ couplings between Co atoms. In order to verify that, simulations with three layers (Pd/Co/Pd) were performed and we observed the same outcome compared to the simulation of only one Co layer (data not shown).

In order to construct the phase diagram presented in Fig. 4 from the main text, as in the IM (case (b)) the Co DMI is zero for symmetry reasons[1], the chosen set of parameters

[1] In real epitaxial symmetric Pd/Co/Pd systems, a DMI can also appear because of elastic strains at the

is from the SIG calculation (case (a)). The experimental anisotropy ranges found in the literature are indicated in Fig. 4 from the main text, where the upper and lower boundary for the sputtering and MBE/e-beam evaporation deposition methods were chosen according to the works from Ref.[35] and Ref.,[36] respectively. We used the 4-IIS approach to obtain the ground state. Another phase diagram, using SA, were also constructed, where it was possible to identify the metastable states. Here, K_i was varied from 0 to 0.14 meV/atom and the scalar DMI factor, ϵ^{DMI}, was varied from 0 to 18 ($i.e.$, $\epsilon^{\mathrm{DMI}} \in [0, 18]$), where the new re-scaled DMI strength is given by

$$\vec{D}_{ij}^{\text{re-scaled}} = \epsilon^{\mathrm{DMI}} \vec{D}_{ij}^{\text{pristine SIG}}. \tag{5}$$

We have shown that the Co-Co DMI strength is affected locally by presence of Pd defects. Thus, here, we consider an approximation where the DMI is re-scaled for all Co-Co i-j interactions. We expect that this approximation is valid in the limit in which there are a sufficient number of interdiffused defects in the sample. In this model, the DMI vector directions are kept unchanged from the original computed ones for the pristine SIG.

In the following section, we discuss some results in more detail.

II. Results

A. Magnetic moments

The magnetic μ_s and orbital μ_l moments (in μ_B) for the Pd and Co atoms, in the SIG and IM, are presented in Fig. 2. Comparing both approaches, we note that the moments for the Co and Pd atoms are very similar at the interface. However, since the Co monolayer is repeated in the IM, the induced magnetic moment (μ_{Pd}) is large in all Pd layers (varying from $\sim 0.2 - 0.4\ \mu_B$), while, in the SIG, the μ_{Pd} decreases and goes to zero as the distance

Pd/Co interface,[34] but this is not the case in our model.

between the Pd and Co layers increases. Then, we verified that the electronic structure of the interface Pd/Co/Pd in the SIG and IM is almost the same, providing a good description of the system. The values obtained here for both spin and orbital magnetic moments are in excellent agreement with theoretical results in the literature.[37] Also, the spin moments per atom obtained by calculations with QE, in all 3 considered structure models, are consistent with the IM values in Fig. 2: $\sim 2.1\,\mu_B$ for Co and $\sim 0.35\,\mu_B$ for the first adjacent Pd layers.

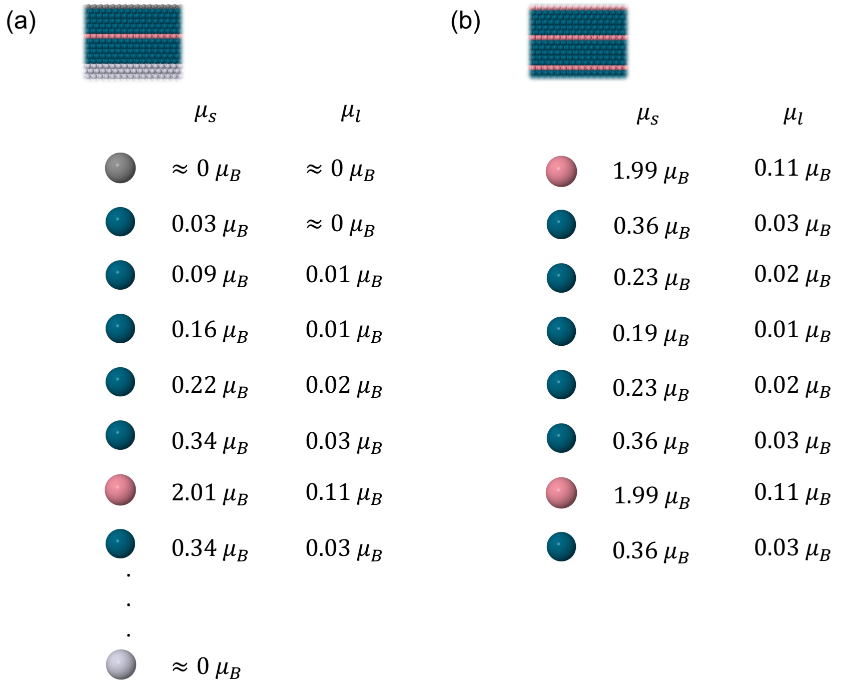

Figure 2: Magnetic (μ_s) and orbital (μ_l) moments for the Pd and Co atoms in the Pd/Co/Pd, calculated as (a) semi-infinite geometry and (b) infinite multilayers. The dark gray, blue, pink and light gray illustrate the empty spheres, Pd, Co and Pd bulk atoms, respectively.

The spin magnetic moments in the systems with defects are presented in Fig. 3. Comparing with the pristine SIG results (Fig. 2(a)), we see that there is a slightly larger induced μ_s in the Pd defects comparing with the Pd atom at the pristine Pd/Co/Pd interface. However, since the Co magnetic moments are not significantly affected by the presence of defects, it can be inferred that the DMI enhancement comes mainly from the symmetry breaking rather than from hybridization effects in the non-magnetic layer.

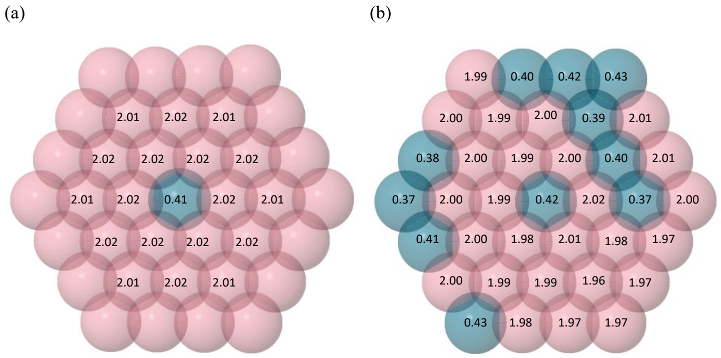

Figure 3: Spin magnetic moments (μ_s) for Co and Pd atoms in the clusters with (a) a single defect and (b) several defects at the Co monolayer.

B. Exchange and DMI

Concerning the pristine exchange coupling results in Fig. 1 from the main text, we note a strong ferromagnetic ($J_{ij} > 0$) nearest-neighbor (NN) interaction for the Co-Co interactions, with very small anti-ferromagnetic ($J_{ij} < 0$) interaction for larger distances. The coupling abruptly decreases for Co-Pd NN pairs and is almost zero for Pd-Pd interactions. Besides that, the J_{ij} values rapidly go to zero for larger distances. In Fig. 1(b) from the main text, we see that the NN DMI strength is much smaller than its respective NN J_{ij}. For further distances the $|\vec{D}_{ij}|$ couplings present oscillations (unlike the exchange interaction), where the coupling for larger distances can be as relevant as the NN interactions. This type of behavior can be correlated to symmetry reasons and it has already been reported in the literature.[38,39] Interestingly, the SIG and IM Co-Pd interactions present similar behavior, except for the NN coupling, which is slightly larger in the IM compared to the SIG.

We note here that an ABCA-B-ABC stacking (trigonal prismatic coordination of Co) has also been considered for the IM. Opposed to the ABC-ABC stacking, the DMI is non-null due to the lack of inversion symmetry. However, its strength is only slightly larger (∼0.04 meV for NN Co-Co coupling) than the one found for the pristine SIG and the $\vec{D}_{ij}$ is almost completely perpendicular to the Co monolayer plane. In order to investigate the ground state magnetic configuration, we have performed spin dynamics simulations for this

specific stacking and we only found monodomain ferromagnetic states. Considering the same approximation of the scaling factor used in the paper (with $\epsilon^{\mathrm{DMI}} = 12$), although it is possible to obtain noncollinear metastable states, the ground state is still monodomain, differently than the ABC stacking case (SIG calculation) which is formed by spin spirals for this specific ϵ^{DMI} (see Fig. 4 from the main text). These results can be explained by the combination of a weak DMI strength and a large out-of-plane component, which is not favorable to the existence of skyrmions (as shown in other works[39,40]). Therefore, although this stacking could be a source of DMI in the infinite multilayer by the global breaking of symmetry, its strength and calculated directions are not sufficient, alone, to account for the existence of chiral spin structures in the experimental sample and it would still need the effect of interdiffusion, as investigated.

In Fig. 4, we present the DMI strength for Co-Co couplings in the system with a single defect. For a fixed Co atom as a nearest-neighbor (NN) of the defect (Fig. 4(a), same as Fig. 2(b) in the main text), the order of magnitude of the DMI strength is ten times larger compared to fixed Co atoms as next nearest-neighbor (NNN) in Fig. 4(b) and as a next-to-next nearest-neighbor (NNNN) in Fig. 4(c). Here, we note that the defect and fixed Co positions define a symmetry axis (represented as a dashed line in Fig. 4), where the Co-Co couplings which are symmetric to this axis present the same DMI strength for most cases. Then, differently than the pristine system, due to the lack of symmetry in the single defect case, it is not possible to associate a unique DMI strength to the distance between atoms i and j, since the couplings depends on the relative position of the Co-Co pair to the defect. We have also verified that most of these symmetric couplings follow the rule $D^z_{ij} = -D^z_{ji}$.

Analogously, we present the Co-Co DMI strength in the system with several defects for fixed Co atoms with one (Fig. 5(a)), two (Fig. 5(b)) and three (Fig. 5(c)) defects atoms as NN. The DMI behavior follows the same trend observed for the system with one defect, where a few Co-Co couplings have a large DMI strength. However, here, no direct correlation was found between the amount of NN defects and the DMI strength.

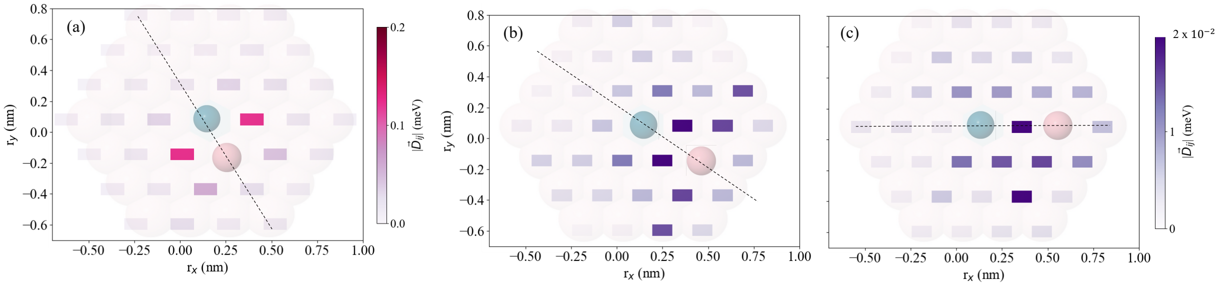

Figure 4: Co-Co DMI strength in the Co monolayer (r_x and r_y) with a single defect (blue sphere) for the cases where the fixed Co atom (pink sphere) is a (a) NN, (b) NNN and (c) NNNN to the defect. The pink and purple colormaps represent the DMI strength of the fixed Co atom with the Co atom in each square site. We present the colormap in purple to highlight the different order of magnitude of DMI strength in cases (b) and (c). Here, the dashed black line is a guide to the eyes and represents a symmetry axis for the interactions.

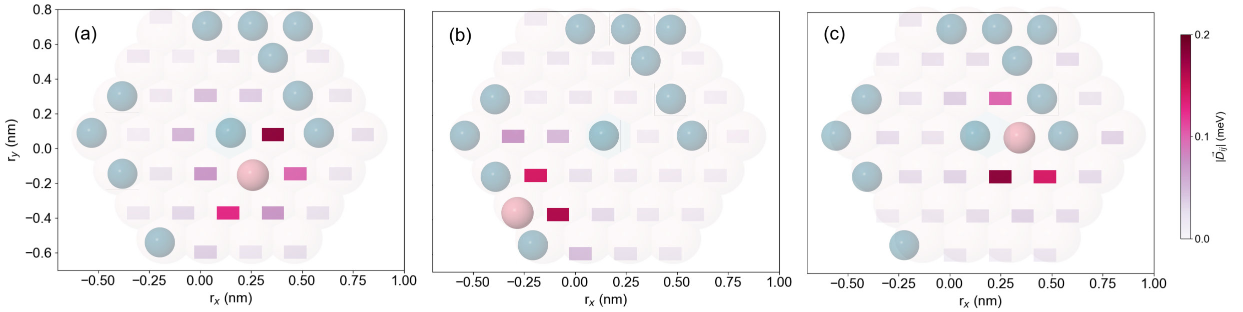

Figure 5: Co-Co DMI strength in the Co monolayer (r_x and r_y) with several defects (blue spheres), where the fixed Co atom (pink spheres) has (a) one (b) two or (c) three defect atoms as NN. The pink colormap represents the DMI strength of the fixed Co atom with the Co atom in each square site.

Fig. 6 shows the Co-Pd coupling for the system with several defects. As expected, the Co-Pd couplings have a smaller DMI strength compared to the Co-Co couplings, since $\mu_{Pd} \ll \mu_{Co}$.

Fig. 7 presents a front and side view of the DMI vectors, considering different positions for fixed Co atoms (A, B, C and D), in the system with several defects. Interestingly, although the DMI vectors do not seem to present a preferential direction with a sense of rotation as in the pristine system, we verified that most Co-Co couplings show a large projected DMI in-plane contribution (Fig. 2 from the main text). Also, the vectors follow the Moriya's symmetry rules,[41,42] where the DMI vectors are perpendicular (within a small error) to the

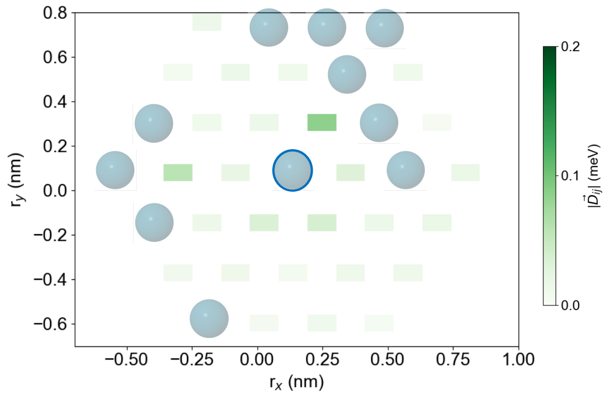

Figure 6: Pd-Co DMI strength in the Co monolayer (r_x and r_y) with several defects. The blue spheres represent the Pd defects and the blue circle denotes the fixed Pd atom. The green colormap represents the DMI strength of the fixed Pd atom with the Co atoms in each square site.

interatomic bond axis between sites i and j.

In Fig. 8, we plot the NN DMI components and norm for fixed Co atoms in positions A, B, C and D, as defined in Fig. 7, where each NN atom is labeled from I to VI. As expected, a larger DMI norm is observed for Co-Co couplings compared to Co-Pd. Besides that, a large D_z component is obtained for a few couplings.

In order to better understand the behavior of the DMI vector components, we plot D_x, D_y and D_z in Fig. 9(a)-(c), respectively, for the single defect case, while in Fig. 9(d)-(f) we plot for the several defects case. From these results it is possible to analyze the balance between positive and negative components, since the DMI vectors directions might present a random distribution due to the random arrangement of defects. For the single defect case, the significant interactions come from D_z and Fig. 9(c) shows these contributions with opposite signs. However, for the several defects case, it is not clear if there is a balance between positive and negative DMI components.

It is also important to consider the interactions from Co defect atoms in the Pd layers for the several defects case. In Fig. 10(a)-(c), we plot the DMI strength ($|\vec{D}_{ij}|$), projected in-plane ($|\vec{D}_{xy}|$) and out-of-plane ($|\vec{D}_z|$) contributions, respectively, for Co-Co and Co-Pd intralayer couplings. Due to the small Pd induced magnetic moment, the Co-Co interactions are generally much larger than Co-Pd ones. Besides that, we note a large in-plane contri-

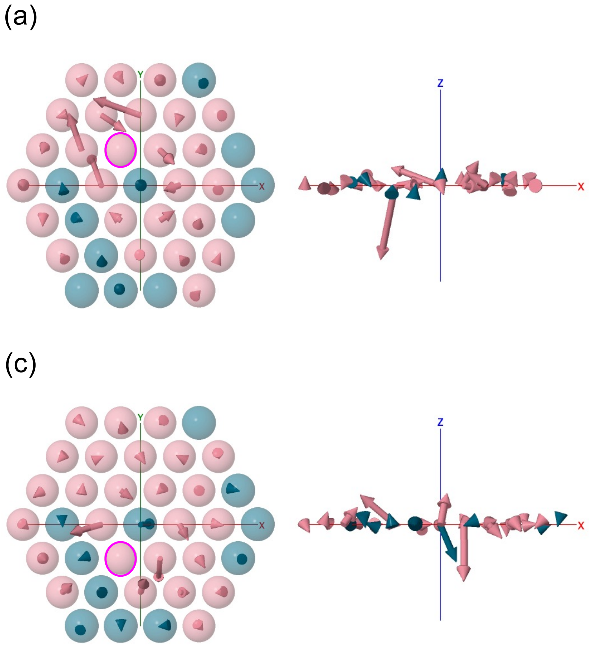

Figure 7: Front and side view of the DMI vectors in the Co monolayer with several defects, considering different fixed Co atoms (pink circle) in different positions, namely: (a) A, (b) B, (c) C and (d) D. The pink (blue) arrows represent the Co-Co (Co-Pd) DMI coupling between the Co atom in the site of the pink circle and the other Co (pink spheres) and Pd (blue spheres) atoms.

bution to the strength of many Co-Co interactions. Similarly to the discussion in the main text for the Co layer (Fig. 2), here we find a possible favorable scenario for the stabilization of skyrmions due to large in-plane DM components.[39,40,43]

In Table 1, we present the exchange interaction, DMI and MAE constant (K) for all the systems calculated here, considering in-plane NN, NNN and NNNN. The values are rounded to the second decimal digit and we note that in the ordered alloy cases, the Co-Co coupling is always intra-layer. Here, the values presented for the single defect case were calculated by considering Co atoms at sites i or j in the first shell of the defect neighbors. It is interesting to see that the DMI can present close NNNN and NNN couplings, as can be seen for the pristine SIG, 50% ordered alloy, and a single defect systems. Surprisingly, in the case of the pristine SIG, the NNNN interaction is similar to the NN coupling. While the exchange interaction is similar to both cases (pristine SIG and IM), for the ordered alloys, the DMI can be one order of magnitude larger compared to the pristine SIG. This indicates that the

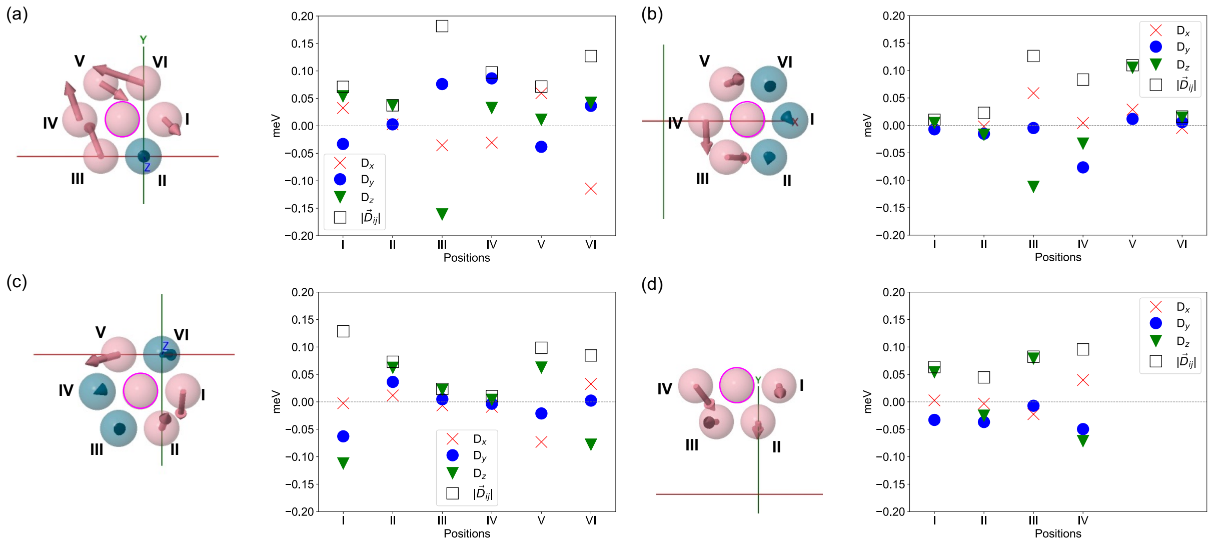

Figure 8: Front view real space representation and plot of the DMI vectors components in the Co monolayer (Co atoms in pink spheres) with several defects (blue spheres) for each nearest-neighbor of the fixed Co atom (pink circle) in different positions, namely: (a) A, (b) B, (c) C and (d) D. The graphs present the components x, y, z and the norm of the DMI vectors.

intermixing of atoms increases the DMI, as shown previously. It is important to highlight here the role of inversion symmetry in the ordered alloys. For the 25% case, the Pd/Co and Co/Pd interfaces are not symmetric, generating a non-null DMI, while for the 50% case, the interfaces are symmetric only for NN Co-Co pairs in the Co monolayer, leading to a null coupling (see Table 1).

We note that other works have reported a non-trivial behavior of the DMI where its strength becomes larger as the number of repetitions of Co/Pd bilayers increases.[34,44] In the context of our work, we show that one of the main sources for the DMI in seemingly symmetric stackings can be the local symmetry breaking caused by defects at the interfaces due to interdiffusion, which experimentally occurs for ultrathin films during the sample deposition (see, e.g., Ref.[45]). Therefore, based on our results, we can conjecture that the increasing number of tri-layers repetitions may lead to a larger occurrence of interdiffusion in the sample, which can contribute to a larger DMI scale. Nevertheless, if that is the case, a future study is necessary in order to verify if there is a sum of these effects.

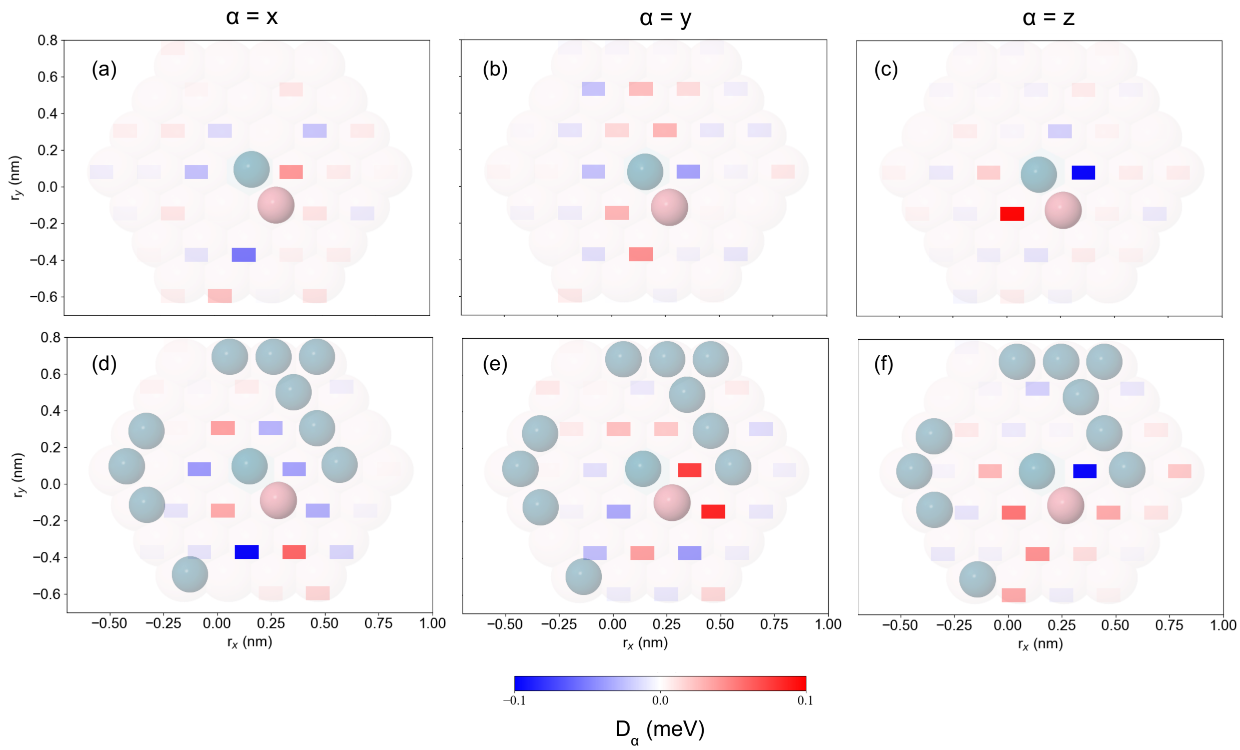

Figure 9: Co-Co DMI components (D_x, D_y and D_z) in the Co monolayer (r_x and r_y) for the cases with: a single defect (a, b, c and d) and several defects (d, e, f and g). The divergent red and blue colormap represents the DMI coupling (squares) between the fixed Co atom (pink) and the Co atom in each site.

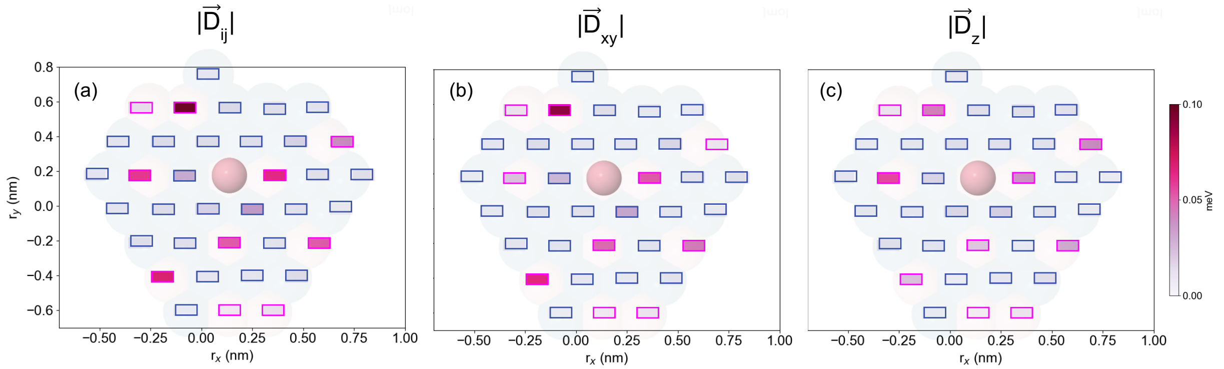

Figure 10: Co-Co and Co-Pd couplings at one of the Pd layers (r_x and r_y), in the several defects case, for: (a) DMI strength ($|\vec{D}_{ij}|$), (b) norm of the projected in-plane DMI vector ($|\vec{D}_{xy}|$) and (c) absolute value of the DMI z-component ($|\vec{D}_z|$). The pink colormap represents the DMI coupling between the fixed Co atom (pink circle) and the Co and Pd atoms in each site. Here, pink (blue) squares are Co-Co (Co-Pd) interactions.

Table 1: Exchange interaction and DMI strength for NN, NNN and NNNN, MAE (K), angle between the easy-axis and xy plane (θ_K), obtained for the different systems studied here: pristine semi-infinite geometry (SIG), pristine infinite multilayers (IM), 25% alloy (a_{25}), 50% alloy (a_{50}), single defect (sd) and several defects (svd). For interactions that present a range of values, we present the minimum, maximum and mean (parenthesis) couplings.

	J_{ij} (meV)		$\lvert\vec{D}_{ij}\rvert$ (meV)			K	θ_K
	NN	NNN	NN	NNN	NNNN	$\frac{meV}{atom}$	
SIG	13.27	1.61	0.01	∼0	0.01	1.14	$\sim 88°$
IM	14.35	1.74	0	0	0	0.23	$\sim 88°$
a_{25}	15.20	2.46	0.04-0.19 (0.08)	0.02-0.05 (0.03)	0.01-0.05 (0.02)	0.44	$\sim 70°$
a_{50}	14.93	2.86	∼0	0-0.08 (0.04)	0.04	0.87	$\sim 23°$
sd	13.43-14.18 (13.97)	1.67-2.05 (1.69)	0.02-0.13 (0.05)	0.08-0.03 (0.01)	0.01-0.02 (0.02)	—	—
svd	11.49-15.71 (12.95)	—	0.01-0.18 (0.09)	—	—	—	—

C. MAE

The MAE of the different systems studied here has also been investigated, since a too strong PMA that favors a ferromagnetic monodomain ground state can suppress spin-spiral and skyrmion structures that otherwise would occur from a finite DMI. On the other hand, a non small PMA can also provide the possibility of metastable domains i.e. magnetic bubbles. Combined with a finite DMI, the bubbles can have properties similar to that of skyrmions forming the so-called skyrmion bubbles (or chiral bubbles).[46,47] The values are described in Table 1 and the total energy as a function of the magnetization angle with the xy plane is presented in Fig. 11. The calculated E_{dip} for the IM is 0.16 meV/atom, which is about a factor of 2 larger than a previous theoretical work,[48] because we consider the Bohr magneton as $\sqrt{2}$ in Rydberg units.[49] The K and θ_K parameters obtained from the fit are presented in

Table 1. As measured in experimental works,[50,51] the uniaxial magnetocrystalline anisotropy of the Pd/Co/Pd multilayers is out-of-plane, and we obtained an easy-axis with an angle of $\sim 90^\circ$ from the surface plane (see Table 1), for both IM and SIG. The MAE in the SIG presents a larger value, which is expected since it is a system with a reduced dimensionality.

In the atomistic calculations, we assume that (as mentioned) only the Co layer contributes to the MAE. In this scenario the conversion to an energy density (MJ/m^3) takes into account the primitive cell around a given Co atom. Thus, to obtain the magnetocrystalline anisotropy, the meV/atom[2] value is divided by the volume $V = \frac{\sqrt{3}a_{2D}^2 t}{2}$, where a_{2D} is the in-plane lattice parameter ($a_{2D} = a_{\mathrm{Pd}}\frac{\sqrt{2}}{2} \sim 2.75\,\text{Å}$) and t is the magnetic layer thickness ($t = n_{\mathrm{layer}}\sqrt{\frac{2}{3}}a_{2D} \sim 2.25\,\text{Å}$; $n_{\mathrm{layer}} = 1$).[52] This definition is valid when no relaxations are taken into account, as it is the case of the pristine systems in RS-LMTO-ASA. In QE ($\vec{k}$-space), however, as the Pd layers participate in the total band energy, the natural way to convert the MAE values is to divide by the volume of the unit cell used in the calculations,[53] which are: 353.18 Å^3 (without relaxation and partial relaxation models) and 335.01 Å^3 (variable cell model). On one hand, with this last definition, we get anisotropies of $\sim 0.7 - 1.7$ MJ/m^3 ($\sim 0.4 - 0.9$ meV/f.u.) from the FT calculations in QE, depending on the model used. This is in good agreement with the theoretical work from Okabayashi *et al.*,[37] which also considered an IM structure for the Pd/1Co/Pd system. On the other hand, the atomistic definition[52,54] used to convert the real-space values (and the y-axis in the phase diagram, Fig. 4, of the main text), resulted in a higher K for the IM in MJ/m^3; the meV/atom value in RS-LMTO-ASA, however, is compatible with the lower limit in QE ($K \sim 0.4$ meV/f.u., which is found for the case without relaxation). With this brief discussion, it is clear that adopting reasonable definitions for each case gives consistent MAE values. Both K results (from QE and RS-LMTO-ASA) are also in good agreement with the interval proposed by Wang *et al.*[55]

The main difference between the pristine Pd/Co/Pd multilayers and the alloys, regarding

[2] The calculated energy for IM in Table 1 in RS-LMTO-ASA is originally a meV/f.u. value, which, with the assumption that only the Co layer contributes to the MAE in the atomistic spin dynamics calculations, is equivalent to a meV/atom unit.

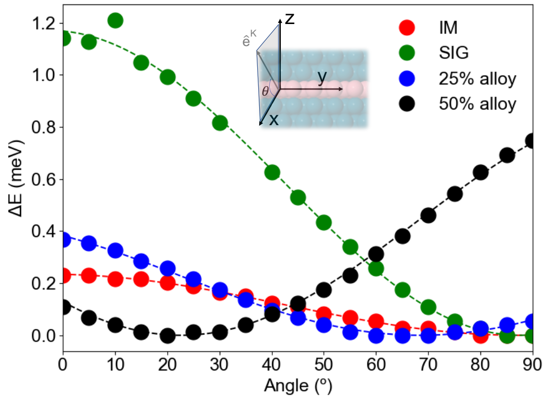

Figure 11: Energy difference as a function of the angle between the magnetization axis and the xy plane for the pristine infinite multilayers, pristine semi-infinite geometry, 25% and 50% alloys. The dots are the energy values calculated with RS-LMTO-ASA method and the dashed line is the fit using Eq. 1. A representation of the coordinate system used here is shown as an inset.

the anisotropy, is the change in the easy-axis direction, where θ_K diminishes and K increases. This indicates that these ordered alloys might not realistically represent the experimental sample with interdiffusion at the Pd/Co/Pd interface. Although we were able to simulate an intermixing of atoms through the alloys, this interdiffusion is ordered, in contrast to what we expect from a sample grown by sputtering. Therefore, an atomic disordered arrangement is essential in order to reproduce experimental results.

D. Curie temperature

To investigate the magnetic behavior of the multilayer at room temperature, we used spin dynamics simulations with finite temperatures. Firstly, we estimated the T_C, i.e., the temperature which occurs a phase transition from an ordered to a disordered magnetic state. For that, the LLG equation (Eq. 3) is solved for different temperatures, in a interval of $0 < T < 700$ K. Since the experimental MAE obtained from sputtered samples can vary up to 0.5 MJ/m^3 (see Fig. 4 from main text), here we considered a value close to the one of Ref.[56] ($K_{\text{eff}} = 0.014$ meV/atom, or $K_{\text{eff}} \sim 0.1$ MJ/m^3 using the atomistic conversion). The normalized magnetization and cumulant, as a function of temperature, are presented in

Fig. 12(a) and Fig. 12(b), respectively. Here, we performed the simulations for lattice sizes of 250 × 250 and 350 × 350, then the T_C found here, using the cumulant crossing method,[57] is estimated to be 311 K. Therefore, at room temperature, the multilayer is very close to the phase transition.

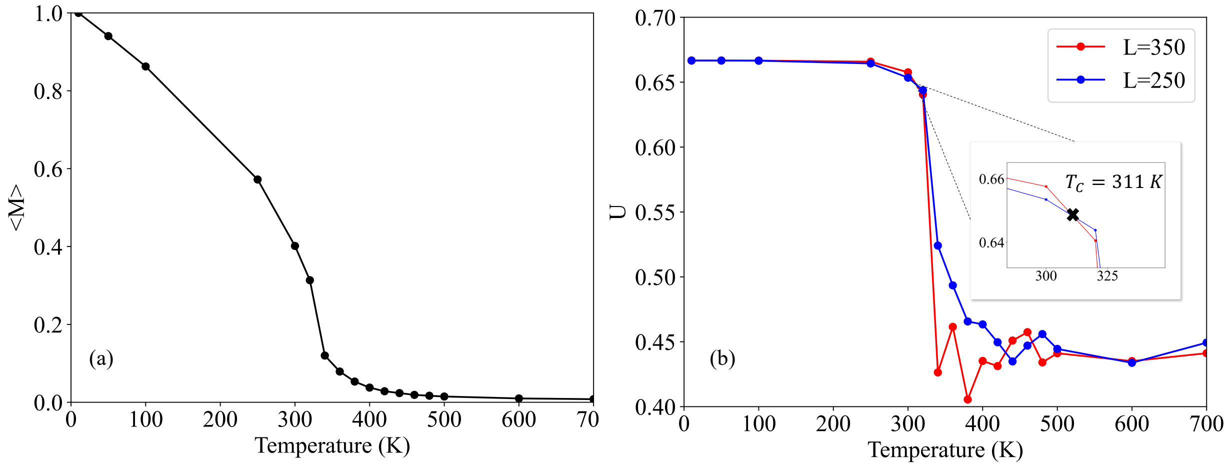

Figure 12: Normalized magnetization (a) and cumulant (b) as a function of temperature, obtained through spin dynamics simulations for the pristine semi-inifinite geometry. The calculations have been done for $L \times L$ lattice sizes, where $L = 250$ in (a) and $L = 250$ or 350 in (b). The inset presents the region where the cumulant lines from both lattice sizes crosses, indicating the Curie temperature (T_C).

To obtain the magnetic moment value for $T = 300$ K, we performed SA, decreasing the temperature from 700 K to 300 K and then using spin dynamics in a simulation of 1.2 ns, with a large damping, to further relax the system. Due to the large thermal effects, the Co layer magnetization fluctuates with a mean value of 0.83 μ_B/atom and a standard deviation of 0.11 μ_B/atom. We have also considered simulations with Pd layers on top/below the Co layer and the magnetization of the Co atom presents a similar behavior. We note here that the comparison between the experimental sum (spin and orbital contributions presented in Fig. 5 from the main text) with the theoretical spin contribution is valid, since the Co orbital magnetic moment from our sum rules is negligible at room temperature.

E. Skyrmions' radius

We have also analyzed the skyrmion profile and its dependence on the scalar DMI factor and MAE. For that, we perform SD in a skyrmionic initial configuration for 500 ps, with different values of K_{eff} and ϵ^{DMI}. In Fig. 13, we present the plot of the skyrmions' radius (R) as a function of ϵ^{DMI}, considering $K_{eff} = 0$ and 0.15 MJ/m^3. The skyrmions' snapshots are also shown. Interestingly, without the addition of anisotropy, the initial configuration is stable ($R \neq 0$) for $\epsilon^{DMI} > 0$. On the other hand, for $K_{eff} = 0.15$ MJ/m^3, the skyrmion is only stable for $\epsilon^{DMI} > 3$. This result indicates that, although the K_{eff} considered here is small, it competes with the DMI favoring monodomain FM states. Besides, it is notable that the skyrmions' radius increase with the scalar DMI factor, as already presented in the literature.[58] The thermal stability of the skyrmion has also been studied and it has shown to be poorly affected by these different DMI factors.

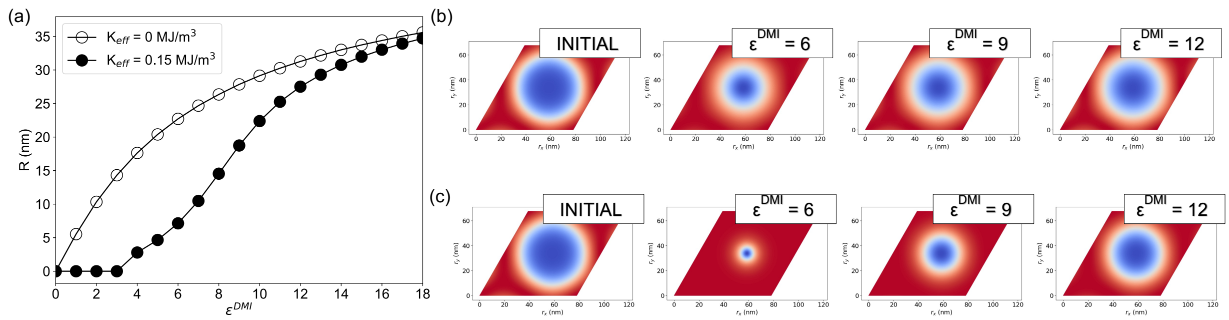

Figure 13: (a) Skymions' radius as a functions of ϵ^{DMI} for different values of PMA calculated in the pristine semi-infinite geometry. Snapshots of the skyrmion in its initial state and after atomistic spin dynamics simulations, using $\epsilon^{DMI} = 6$, 9 and 12, for (b) $K_{eff} = 0$ MJ/m^3 and (c) $K_{eff} = 0.15$ MJ/m^3. The simulations were performed for a 285×285 spin lattice.

F. Metastable states

Finally, in Fig. 14, we show the respective metastable states snapshots obtained with SA for the phase diagram (Fig. 4 in the main text). It is important to highlight that the metastable states calculated here represent one possible configuration among several local minima in the potential energy surface. As mentioned in the main text, metastable stripes

and skyrmionic states can be obtained in the monodomain-FM (m-FM) and spin spiral (SS) regions, respectively. A narrow transition region, between the m-FM and SS phases, with isolated skyrmions can also be seen in Fig. 14. We also notice that even for very large anisotropies, no magnetic bubbles are observed in the simulations. Since this might be a finite-size effect in our atomistic simulations we can however not rule out that magnetic bubbles can occur on larger length scales in the Pd/Co/Pd system.

In order to exemplify the DMI values used to build the phase diagram (Fig. 4 in the main text) and to obtain the metastable states (Fig. 14), in Table 2 we present the values (in meV) used for a few of the scalar DMI factors considered (ϵ^{DMI}). The correspondence between the systems calculated here and ϵ^{DMI} are also indicated. We note that in order to associate the ϵ^{DMI} with a specific system, an approximation is made where only the average and maximum NN couplings, from the single and several defects case, is compared to the NN interactions in Table 2.

Table 2: Co-Co DMI strength (meV) for different values of the scalar DMI factor (ϵ^{DMI}), according to the distance (d) between the Co atoms. The systems corresponding to each ϵ^{DMI} are also indicated (as shown in the phase diagram in Fig. 4 from the main text): semi-infinite geometry (SIG), average NN Co-Co coupling from the single defect (sd) and several defects (svd) cases and maximum NN Co-Co coupling from the sd case.

d (Å)	ϵ^{DMI}			
	1	7	12	16
2.75	0.008	0.055	0.094	0.126
4.76	0.001	0.006	0.011	0.014
5.50	0.011	0.078	0.134	0.178
7.28	0.001	0.008	0.014	0.018
8.25	0.001	0.005	0.009	0.012
system	SIG	sd_{NN}^{mean}	svd_{NN}^{mean}	sd_{NN}^{max}

III. Experimental details

The Pd/Co/Pd multilayers were grown onto SiO_2 substrate by magnetron sputtering with base and deposition pressure of 1×10^{-8} and 3×10^{-8} mbar, respectively. The tri-layer Pd/Co/Pd

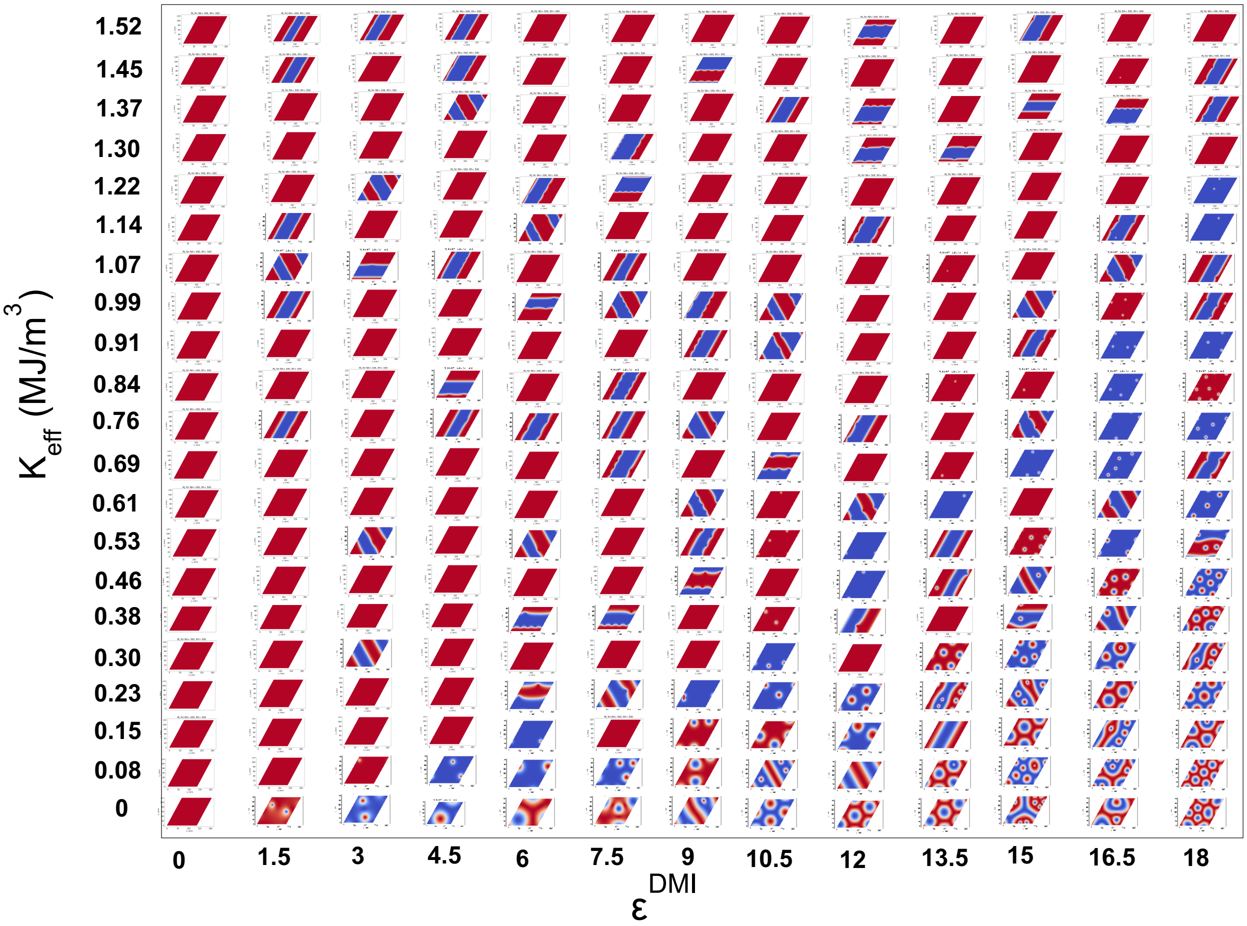

Figure 14: Schematic representation of the corresponding snapshots of metastable states at $T = 10^{-4}$ K and zero external magnetic field, obtained through SA for the Pd/Co/Pd phase diagram (Fig. 4 in the main text). The effective anisotropy strength, $K_{\rm eff}$, and the scalar DMI factor ($\epsilon^{\rm DMI}$) are varied. The $K_{\rm eff}$ values in the y-axis are converted from meV/atom to MJ/m^3 using the atomistic definition (see discussion above).

was repeated 15 times, with Pd thickness of 1 nm and Co thickness of 0.2 nm. We performed X-ray absorption spectroscopy (XAS) and X-ray magnetic circular dichroism (XMCD) at U11 planar grating monochromator (PGM) soft X-ray beamline of the Brazilian Synchrotron Light Laboratory (LNLS).[59] We used a magnetic superconduct and measured the XAS under out-of-plane magnetic field of 0.3 T, parallel to the right and left circularly polarized incoming X-rays.

A. XMCD and XAS measurements

To extract the orbital and spin magnetic moments of Co atoms, and hence the total magnetic moment, we employed L-edge sum rules on the XAS and XMCD acquired for each Co layer by using the following relationships,

$$M_o = \frac{4q}{3r} n_h \tag{6}$$

$$M_{spin} = \frac{6p - 4q}{r} n_h \tag{7}$$

where q, p and r are quantities extracted from the XAS and XMCD spectra, while n_h is the number of empty states above the Fermi energy available for the $2p \rightarrow 3d$ core-level electronic transition. The total magnetic moments (μ) per Co atoms ($M_o + M_s$), were obtained in the sum rules using n_h acquired by first-principles calculations.

References

(1) Frota-Pessôa, S. First-principles real-space linear-muffin-tin-orbital calculations of 3d impurities in Cu. *Phys. Rev. B* **1992**, *46*, 14570–14577.

(2) Peduto, P. R.; Frota-Pessôa, S.; Methfessel, M. S. First-principles linear muffin-tin

orbital atomic-sphere approximation calculations in real space. *Phys. Rev. B* **1991**, *44*, 13283–13290.

(3) Klautau, A. B.; Legoas, S. B.; Muniz, R. B.; Frota-Pessôa, S. Magnetic behavior of thin Cr layers sandwiched by Fe. *Phys. Rev. B* **1999**, *60*, 3421–3427.

(4) Rodrigues, D. C. M.; Szilva, A.; Klautau, A. B.; Bergman, A.; Eriksson, O.; Etz, C. Finite-temperature interatomic exchange and magnon softening in Fe overlayers on Ir(001). *Phys. Rev. B* **2016**, *94*, 014413.

(5) Bezerra-Neto, M. M.; Ribeiro, M. S.; Sanyal, B.; Bergman, A.; Muniz, R. B.; Eriksson, O.; Klautau, A. B. Complex magnetic structure of clusters and chains of Ni and Fe on Pt(111). *Sci. Rep.* **2013**, *3*, 3054.

(6) Cardias, R.; Bezerra-Neto, M. M.; Ribeiro, M. S.; Bergman, A.; Szilva, A.; Eriksson, O.; Klautau, A. B. Magnetic and electronic structure of Mn nanostructures on Ag(111) and Au(111). *Phys. Rev. B* **2016**, *93*, 014438.

(7) Kvashnin, Y. O.; Cardias, R.; Szilva, A.; Di Marco, I.; Katsnelson, M. I.; Lichtenstein, A. I.; Nordström, L.; Klautau, A. B.; Eriksson, O. Microscopic Origin of Heisenberg and Non-Heisenberg Exchange Interactions in Ferromagnetic bcc Fe. *Phys. Rev. Lett.* **2016**, *116*, 217202.

(8) Hohenberg, P.; Kohn, W. Inhomogeneous Electron Gas. *Phys. Rev.* **1964**, *136*, B864–B871.

(9) Haydock, R. The recursive solution of the Schrödinger equation. *Comput. Phys. Commun.* **1980**, *20*, 11 – 16.

(10) Beer, N.; Pettifor, D. G. *The Electronic Structure of Complex Systems*; Springer US, 1984.

(11) von Barth, U.; Hedin, L. A Local Exchange-Correlation Potential for the Spin Polarized Case: I. *J. Phys. C Solid State Phys.* **1972**, *5*, 1629.

(12) Liechtenstein, A. I.; Katsnelson, M. I.; Antropov, V. P.; Gubanov, V. A. Local spin density functional approach to the theory of exchange interactions in ferromagnetic metals and alloys. *J. Magn. Magn. Mater.* **1987**, *67*, 65 – 74.

(13) Cardias, R.; Bergman, A.; Szilva, A.; Kvashnin, Y. O.; Fransson, J.; Klautau, A. B.; Eriksson, O.; Nordström, L. First-principles Dzyaloshinskii–Moriya interaction in a non-collinear framework. *Sci. Rep.* **2020**, *10*, 20339.

(14) Yang, H.; Liang, J.; Cui, Q. First-principles calculations for Dzyaloshinskii–Moriya interaction. *Nat. Rev. Phys.* **2022**, 1–19.

(15) Giannozzi, P. et al. QUANTUM ESPRESSO: a modular and open-source software project for quantum simulations of materials. *Journal of Physics: Condensed Matter* **2009**, *21*, 395502.

(16) Giannozzi, P. et al. Advanced capabilities for materials modelling with Quantum ESPRESSO. *Journal of Physics: Condensed Matter* **2017**, *29*, 465901.

(17) Li, D.; Barreteau, C.; Castell, M. R.; Silly, F.; Smogunov, A. Out- versus in-plane magnetic anisotropy of free Fe and Co nanocrystals: Tight-binding and first-principles studies. *Phys. Rev. B* **2014**, *90*, 205409.

(18) Perdew, J. P.; Burke, K.; Ernzerhof, M. Generalized Gradient Approximation Made Simple. *Phys. Rev. Lett.* **1996**, *77*, 3865–3868.

(19) Perdew, J. P.; Ruzsinszky, A.; Csonka, G. I.; Vydrov, O. A.; Scuseria, G. E.; Constantin, L. A.; Zhou, X.; Burke, K. Restoring the Density-Gradient Expansion for Exchange in Solids and Surfaces. *Phys. Rev. Lett.* **2008**, *100*, 136406.

(20) Monkhorst, H. J.; Pack, J. D. Special points for Brillouin-zone integrations. *Phys. Rev. B* **1976**, *13*, 5188–5192.

(21) Marzari, N.; Vanderbilt, D.; De Vita, A.; Payne, M. C. Thermal Contraction and Disordering of the Al(110) Surface. *Phys. Rev. Lett.* **1999**, *82*, 3296–3299.

(22) Legoas, S. B.; Araujo, A. A.; Laks, B.; Klautau, A. B.; Frota-Pessôa, S. Self-consistent electronic structure of Mo(001) and W(001) surfaces. *Phys. Rev. B* **2000**, *61*, 10417–10426.

(23) Costa, A. T.; Muniz, R. B.; Lounis, S.; Klautau, A. B.; Mills, D. L. Spin-orbit coupling and spin waves in ultrathin ferromagnets: The spin-wave Rashba effect. *Phys. Rev. B* **2010**, *82*, 014428.

(24) Antropov, V. P.; Katsnelson, M. I.; Harmon, B. N.; van Schilfgaarde, M.; Kusnezov, D. Spin dynamics in magnets: Equation of motion and finite temperature effects. *Physical Review B* **1996**, *54*, 1019–1035.

(25) Eriksson, O.; Bergman, A.; Bergqvist, L.; Hellsvik, J. *Atomistic Spin Dynamics: Foundations and Applications*; Oxford University Press: Oxford, 2017; p 272.

(26) Skubic, B.; Hellsvik, J.; Nordström, L.; Eriksson, O. A method for atomistic spin dynamics simulations: implementation and examples. *Journal of Physics: Condensed Matter* **2008**, *20*, 315203.

(27) Lin, S.-Z.; Hayami, S. Ginzburg-Landau theory for skyrmions in inversion-symmetric magnets with competing interactions. *Phys. Rev. B* **2016**, *93*, 064430.

(28) Rózsa, L.; Palotás, K.; Deák, A.; Simon, E.; Yanes, R.; Udvardi, L.; Szunyogh, L.; Nowak, U. Formation and stability of metastable skyrmionic spin structures with various topologies in an ultrathin film. *Phys. Rev. B* **2017**, *95*, 094423.

(29) Wang, W.; Machta, J.; Katzgraber, H. G. Comparing Monte Carlo methods for finding ground states of Ising spin glasses: Population annealing, simulated annealing, and parallel tempering. *Phys. Rev. E* **2015**, *92*, 013303.

(30) Yang, X.-S. In *Nature-Inspired Optimization Algorithms (Second Edition)*, second edition ed.; Yang, X.-S., Ed.; Academic Press, 2021; pp 91–100.

(31) Xu, Q.; Shen, Z.; Pereiro, M.; Herman, P.; Eriksson, O.; Delin, A. Genetic-tunneling driven energy optimizer for magnetic system. *arXiv:2301.00207* **2022**,

(32) Gilbert, T. A phenomenological theory of damping in ferromagnetic materials. *IEEE Transactions on Magnetics* **2004**, *40*, 3443–3449.

(33) Slonczewski, J. C. Current-driven excitation of magnetic multilayers. *Journal of Magnetism and Magnetic Materials* **1996**, *159*, L1–L7.

(34) Davydenko, A. V.; Kozlov, A. G.; Stebliy, M. E.; Kolesnikov, A. G.; Sarnavskiy, N. I.; Iliushin, I. G.; Golikov, A. P. Dzyaloshinskii-Moriya interaction and chiral damping effect in symmetric epitaxial Pd/Co/Pd(111) trilayers. *Phys. Rev. B* **2021**, *103*, 094435.

(35) Takahashi, H.; Fukatsu, S.; Tsunashima, S.; Uchiyama, S. Perpendicular magnetic anisotropy of Pd/Co- and Pd/Ni-multilayers. *Journal of Magnetism and Magnetic Materials* **1992**, *104-107*, 1831–1832.

(36) Davydenko, A. V.; Kozlov, A. G.; Ognev, A. V.; Stebliy, M. E.; Samardak, A. S.; Ermakov, K. S.; Kolesnikov, A. G.; Chebotkevich, L. A. Origin of perpendicular magnetic anisotropy in epitaxial Pd/Co/Pd(111) trilayers. *Physical Review B* **2017**, *95*, 064430.

(37) Okabayashi, J.; Miura, Y.; Munekata, H. Anatomy of interfacial spin-orbit coupling in Co/Pd multilayers using X-ray magnetic circular dichroism and first-principles calculations. *Scientific Reports* **2018**, *8*, 8303.

(38) Sadhukhan, B.; Bergman, A.; Kvashnin, Y. O.; Hellsvik, J.; Delin, A. Spin-lattice couplings in two-dimensional CrI_3 from first-principles computations. *Phys. Rev. B* **2022**, *105*, 104418.

(39) Carvalho, P. C.; Miranda, I. P.; Klautau, A. B.; Bergman, A.; Petrilli, H. M. Complex magnetic textures in Ni/Ir_n/Pt(111) ultrathin films. *Phys. Rev. Materials* **2021**, *5*, 124406.

(40) Miranda, I. P.; Klautau, A. B.; Bergman, A.; Petrilli, H. M. Band filling effects on the emergence of magnetic skyrmions: Pd/Fe and Pd/Co bilayers on Ir(111). *Phys. Rev. B* **2022**, *105*, 224413.

(41) Moriya, T. New Mechanism of Anisotropic Superexchange Interaction. *Phys. Rev. Lett.* **1960**, *4*, 228–230.

(42) Crépieux, A.; Lacroix, C. Dzyaloshinsky–Moriya interactions induced by symmetry breaking at a surface. *J. Magn. Magn. Mater.* **1998**, *182*, 341 – 349.

(43) Kuepferling, M.; Casiraghi, A.; Soares, G.; Durin, G.; Garcia-Sanchez, F.; Chen, L.; Back, C. H.; Marrows, C. H.; Tacchi, S.; Carlotti, G. Measuring interfacial Dzyaloshinskii-Moriya interaction in ultrathin magnetic films. *Rev. Mod. Phys.* **2023**, *95*, 015003.

(44) Pollard, S. D.; Garlow, J. A.; Yu, J.; Wang, Z.; Zhu, Y.; Yang, H. Observation of stable Néel skyrmions in cobalt/palladium multilayers with Lorentz transmission electron microscopy. *Nature Communications* **2017**, *8*, 14761.

(45) Verna, A.; Alippi, P.; Offi, F.; Barucca, G.; Varvaro, G.; Agostinelli, E.; Albrecht, M.; Rutkowski, B.; Ruocco, A.; Paoloni, D.; Valvidares, M.; Laureti, S. ACS Applied Materials & Interfaces. **2022**, *14*, 1944.

(46) Jiang, W.; Upadhyaya, P.; Zhang, W.; Yu, G.; Jungfleisch, M. B.; Fradin, F. Y.; Pearson, J. E.; Tserkovnyak, Y.; Wang, K. L.; Heinonen, O.; te Velthuis, S. G. E.; Hoffmann, A. Blowing magnetic skyrmion bubbles. *Science* **2015**, *349*, 283–286.

(47) Bogatyrëv, A. B.; Metlov, K. L. What makes magnetic skyrmions different from magnetic bubbles? *Journal of Magnetism and Magnetic Materials* **2018**, *465*, 743–746.

(48) Daalderop, G. H. O.; Kelly, P. J.; Schuurmans, M. F. H. First-principles calculation of the magnetic anisotropy energy of $(Co)_n/(X)_m$ multilayers. *Phys. Rev. B* **1990**, *42*, 7270–7273.

(49) Tung, J. C.; Guo, G. Y. Systematic ab initio study of the magnetic and electronic properties of all 3*d* transition metal linear and zigzag nanowires. *Phys. Rev. B* **2007**, *76*, 094413.

(50) Carcia, P. F.; Meinhaldt, A. D.; Suna, A. Perpendicular magnetic anisotropy in Pd/Co thin film layered structures. *Applied Physics Letters* **1985**, *47*, 178–180.

(51) Draaisma, H.; de Jonge, W.; den Broeder, F. Magnetic interface anisotropy in Pd/Co and Pd/Fe multilayers. *Journal of Magnetism and Magnetic Materials* **1987**, *66*, 351–355.

(52) Simon, E.; Rózsa, L.; Palotás, K.; Szunyogh, L. Magnetism of a Co monolayer on Pt(111) capped by overlayers of 5*d* elements: A spin-model study. *Phys. Rev. B* **2018**, *97*, 134405.

(53) Miura, Y.; Ozaki, S.; Kuwahara, Y.; Tsujikawa, M.; Abe, K.; Shirai, M. The origin of perpendicular magneto-crystalline anisotropy in L10–FeNi under tetragonal distortion. *J. Phys.: Condens. Matter* **2013**, *25*, 106005.

(54) Jia, H.; Zimmermann, B.; Hoffmann, M.; Sallermann, M.; Bihlmayer, G.; Blügel, S.

Material systems for FM-/AFM-coupled skyrmions in Co/Pt-based multilayers. *Phys. Rev. Mater.* **2020**, *4*.

(55) Wang, D.-s.; Wu, R.; Freeman, A. J. Magnetocrystalline anisotropy of Co-Pd interfaces. *Physical Review B* **1993**, *48*, 15886–15892.

(56) Brandão, J.; Dugato, D. A.; Seeger, R. L.; Denardin, J. C.; Mori, T. J. A.; Cezar, J. C. Observation of magnetic skyrmions in unpatterned symmetric multilayers at room temperature and zero magnetic field. *Scientific Reports* **2019**, *9*, 4144.

(57) Landau, D. P.; Binder, K. *A Guide to Monte Carlo Simulations in Statistical Physics*, 4th ed.; Cambridge University Press, 2014.

(58) Behera, A. K.; Mishra, S. S.; Mallick, S.; Singh, B. B.; Bedanta, S. Size and shape of skyrmions for variable Dzyaloshinskii–Moriya interaction and uniaxial anisotropy. *Journal of Physics D: Applied Physics* **2018**, *51*, 285001.

(59) Cezar, J. C. et al. The U11 PGM beam line at the Brazilian National Synchrotron Light Laboratory. *Journal of Physics: Conference Series* **2013**, *425*, 072015.
